# A translocation-competent pore is required for *Shigella flexneri* to escape from the double membrane vacuole during intercellular spread

**DOI:** 10.1128/mbio.01674-25

**Published:** 2025-08-11

**Authors:** Julie E. Raab, Tucker B. Harju, Jody D. Toperzer, Jeffrey K. Duncan-Lowey, Connon I. Thomas, Anza Darehshouri, Marcia B. Goldberg, Brian C. Russo

**Affiliations:** 1 Department of Immunology and Microbiology, University of Colorado—Anschutz Medical Campus 549224, Aurora, Colorado, USA; 2 Division of Infectious Diseases, Massachusetts General Hospital 2348, Boston, Massachusetts, USA; 3 Department of Microbiology, Blavatnik Institute, Harvard Medical School 630058 , Boston, Massachusetts, USA; 4 Department of Cell and Developmental Biology, University of Colorado—Anschutz Medical Campus 233177, , Aurora, Colorado, USA; University of Pretoria, Pretoria, Gauteng, South Africa

**Keywords:** *Shigella*, type 3 secretion, IpaC, intercellular spread, translocation, double membrane vacuole

## Abstract

Type 3 secretion systems (T3SSs) enable bacterial virulence by translocating virulence proteins (effectors) into host cells. *Shigella flexneri* requires T3SS to invade and to spread between cells in the colon. To spread, *S. flexneri* forms membrane protrusions that push into the adjacent host cell. These protrusions are resolved into double membrane vacuoles (DMVs) that the bacteria quickly escape. The mechanisms required for escape from the DMV are poorly understood, but the T3SS translocon pore protein IpaC is essential. Here, we show IpaC forms a pore that is competent for the translocation of T3SS effectors as bacteria spread between cells. To do so, we used a genetic approach to test mutations of IpaC that disrupt its ability to translocate and to form pores. We show that during spread, IpaC is efficiently inserted into the plasma membrane, the membrane-embedded IpaC forms pore complexes, and the IpaC-dependent pores translocate effectors that are necessary for *S. flexneri* to escape the DMV. We show that T3SS activation is regulated through a distinct mechanism at spread compared to invasion; activation of T3SS secretion does not require pore formation during spread. We further show that IpaC enables a sequential breakdown of the membranes of the DMV. Thus, we show that a distinct regulation of the T3SS during *S. flexneri* intercellular spread enables the placement of effectors both around *S. flexneri* and across membranes of the DMV. Altogether, this study provides new insights into how *S. flexneri* escapes the DMV.

**IMPORTANCE:**

The type 3 secretion system (T3SS) is required for virulence inmany bacterial pathogens that infect humans. The T3SS forms a pore through which virulence proteins are delivered into host cells, enabling bacterial infection. Our work investigates the *Shigella* translocon pore protein IpaC, which is essential not only for bacteria to invade cells but also for bacteria to spread between cells. The ability to spread between cells is essential for pathogenesis; thus, understanding the mechanisms that enable spread is important for determining how *S. flexneri* infection causes illness. We show that IpaC delivers virulence factors across the host membrane for *S. flexneri* to efficiently spread. This study furthers our understanding of the mechanisms involved in T3SS secretion and of translocon pore function during *S. flexneri* intercellular spread.

## INTRODUCTION

*Shigella flexneri* is a gram-negative bacterial pathogen and a major cause of moderate-to-severe diarrheal illness in humans (1). Disease arises from invasion of and spread between epithelial cells in the colonic epithelium. Intercellular spread causes death of more epithelial cells than invasion alone, and this disruption of the intestinal barrier increases colonic inflammation (2–4). Thus, the ability to spread is essential for disease progression to severe, bloody diarrhea (5, 6). Both invasion and spread require a type 3 secretion system (T3SS). The T3SS is a needle-like structure that delivers virulence proteins (effectors) into epithelial cells. The delivered effectors reprogram cellular pathways that are necessary for *S. flexneri* to establish an intracellular niche.

To access the host cytosol during invasion, the effectors of *S. flexneri* are delivered across the plasma membrane through a pore, known as the translocon pore. This pore is generated by T3SS-mediated delivery of the bacterial proteins IpaC and IpaB into the plasma membrane (7). The translocon pore protein IpaC then interacts with host cytosolic intermediate filaments to activate secretion of effectors through the T3SS into the host cell (8, 9). Thus, at invasion, IpaC forms a pore that regulates the secretion of effector proteins through the T3SS. In contrast to invasion, intermediate filaments are dispensable to activate T3SS secretion when *S. flexneri* spreads (10). IpaC enables cytosolic *S. flexneri* to form protrusions in the host membrane by releasing tension at the membrane through its interaction with β-catenin (10, 11), a cytosolic protein, and component of adherens junctions (12, 13). The protrusion extends into and is engulfed by the neighboring cell (14). The resolution of the protrusion results in the formation of a double membrane vacuole (DMV) containing membranes from the donor and the recipient cell. Bacteria escape from the DMV into the cytosol of the neighboring cell. Whereas mutations of IpaC that specifically disrupt its interaction with β-catenin reduce the efficiency of spread by limiting protrusion formation (10), the complete loss of IpaC prevents spread (7, 15). When IpaC is induced during invasion but not induced during spread, bacteria are observed as trapped in the DMV (15, 16), suggesting that escape from the DMV requires IpaC activity beyond its role in the release of actomyosin-derived tension at the membrane.

The objective of this study was to determine the functional role of IpaC in *S. flexneri* to escape from the DMV. We show that, in the absence of IpaC, secretion of type 3 effectors was efficient, but more bacteria were retained in DMVs during intercellular spread. To assess how IpaC contributes to escape from the DMV, we developed an inducible system that enabled the study of IpaC variants with specific functional defects. Using this system, we found that the formation of T3SS translocation-competent pores was required for bacteria to spread. Whereas disruption of the donor membrane of the DMV is facilitated by pore formation, disruption of the recipient membrane requires pores that are competent for effector translocation. Overall, these data show that for *S. flexneri* to escape from DMVs and spread intercellularly, it must produce a pore at the time of spread that is competent for translocation of effectors.

## RESULTS

### IpaC and IpaB are present in the host membrane at the time of bacterial spread

**Fig 1:**
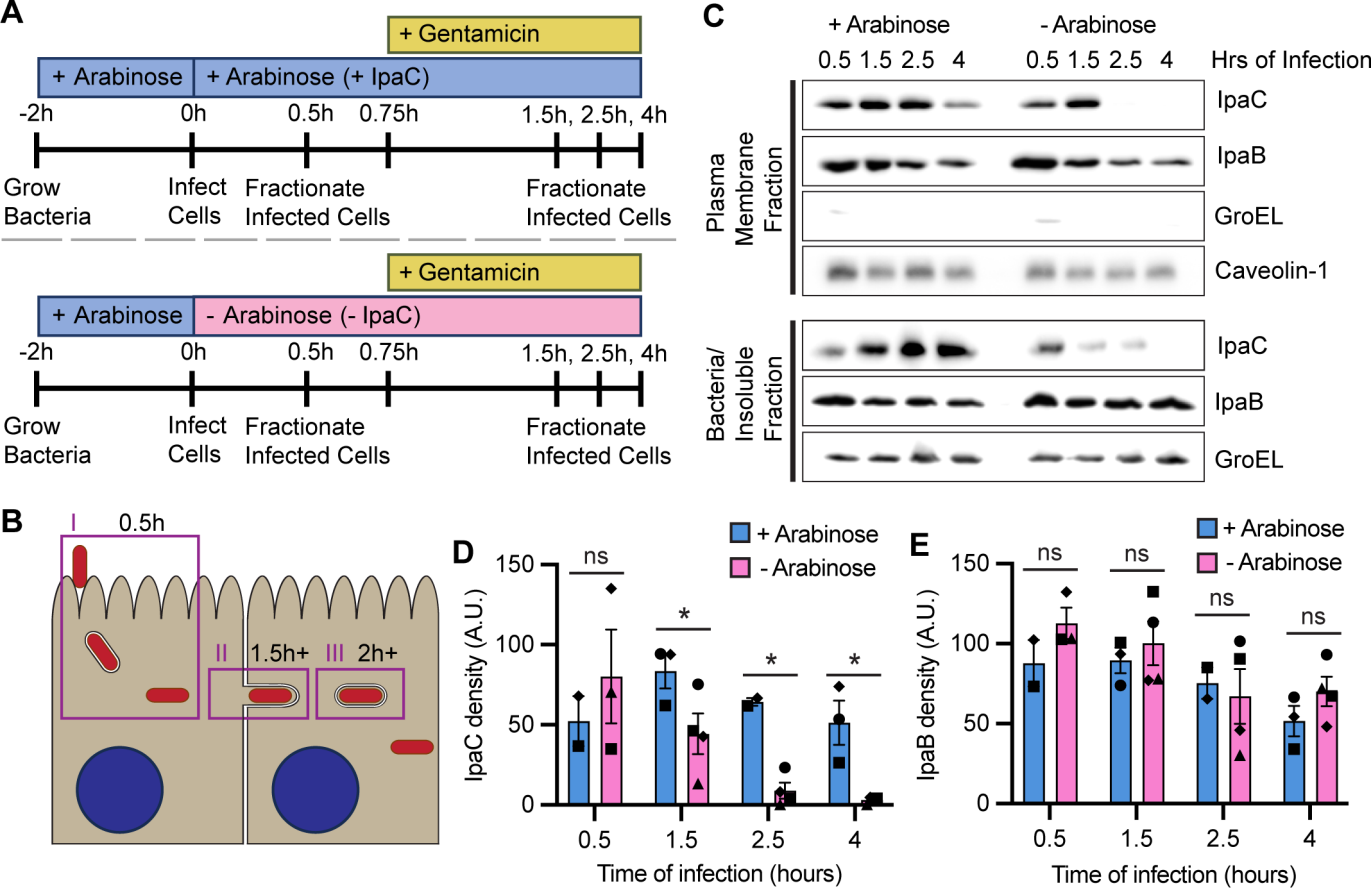
IpaC and IpaB are present in the host membrane at the time of bacterial spread. (**A**) Schematic showing the experimental protocol. + or – IpaC indicates whether arabinose was present in the cell culture media to induce or not induce the production of IpaC throughout infection. Gentamicin was added at 45 minutes of infection to kill extracellular bacteria. (**B**) Schematic illustrating when stages of bacterial invasion and intercellular spread begin: (I) invasion and escape from the entry vacuole occur within 0.5 hours, (II) protrusions begin around 1.5 hours, and (III) DMVs form within 2 hours of infection. (**C**) Representative western blots showing abundance of IpaC, IpaB, GroEL (bacterial cytoplasmic protein), and caveolin-1 (eukaryotic membrane protein) in the membrane fraction (top panels) and abundance of IpaC, IpaB, and GroEL in the insoluble fraction/bacteria (bottom panels). (**D and E**) Quantitative measurements of IpaC (**D**) or IpaB (**E**) in the membrane fraction with (light blue) or without (pink) arabinose included in the media to induce IpaC production. Two to four independent experiments were performed for each infection condition at each timepoint. Data are mean ± SEM; each experiment is matched by symbol. Mixed effects analysis with Fisher’s multiple comparisons test, *P < 0.05.

IpaC is essential for spread (15), during which it enables protrusion formation and escape from DMVs. The function and location of IpaC are well characterized during invasion; IpaC primarily inserts in the host membrane as a component of the translocon pore, and its C-terminal region is on the cytoplasmic side of the plasma membrane (7, 17, 18). To determine whether translocon pore proteins associate with the membrane during spread, we tested whether IpaC and IpaB were localized to the host membrane throughout the course of infection. We infected HeLa cells with *S. flexneri *∆*ipaC* expressing *ipaC* under the control of the arabinose-inducible pBAD promoter (*Sf *∆*ipaC::ipaC*) (Fig. 1A). Bacteria begin to spread around 1.5 hours of infection and resolve into DMVs by 2 hours of infection regardless of whether bacteria produce IpaC or not (15, 16, 19–22) (Fig. 1B). We examined the abundance of our target proteins in the host membrane throughout infection using cellular fractionation; infected cells were fractionated at multiple times between 30 minutes and 4 hours of infection (Fig. 1A). The detergent saponin permeabilized the plasma membrane of the infected cells and released the cytosolic fraction. Solubilization of the membranes by the detergent Triton X-100 enabled recovery of the membrane components. The insoluble components and intact bacteria were removed by centrifugation, as done previously (8–10, 18, 23–25). We found that for IpaC to be present in the membrane at later infection times, it must be continuously produced. In the absence of arabinose, the abundance of IpaC is significantly reduced in the host membrane fraction and from the bacteria by 2.5 hours of infection (Fig. 1C through D). The abundance of IpaB in the membrane was independent of the abundance of IpaC (Fig. 1C and E), which shows either that insertion of IpaB into the membrane at spread is independent of IpaC presence or that IpaB is stably maintained in the membrane from invasion. To exclude the possibility that new invasion events were the source of IpaC and IpaB in the membrane during spread, cells were washed to remove unattached bacteria, and gentamicin was added after 45 minutes of infection to kill remaining extracellular bacteria (Fig. 1A). In these experiments, the absence of the bacterial cytosolic protein GroEL in the plasma membrane fraction indicated that *S. flexneri* was not lysed by our fractionation approach. The eukaryotic plasma membrane protein caveolin-1 showed that the plasma membrane components were localized to the expected fraction. Similar to our observations in HeLa cells, translocon pore proteins localized to the membrane of Caco-2 cells, a human colonic epithelial cell line (Fig. S1). Altogether, these data show that the translocon pore protein IpaC is continuously inserted into the plasma membrane during spread, regardless of cell type.

### IpaC contributes to bacterial escape from DMVs, but effector secretion is activated independent of IpaC

**Fig 2:**
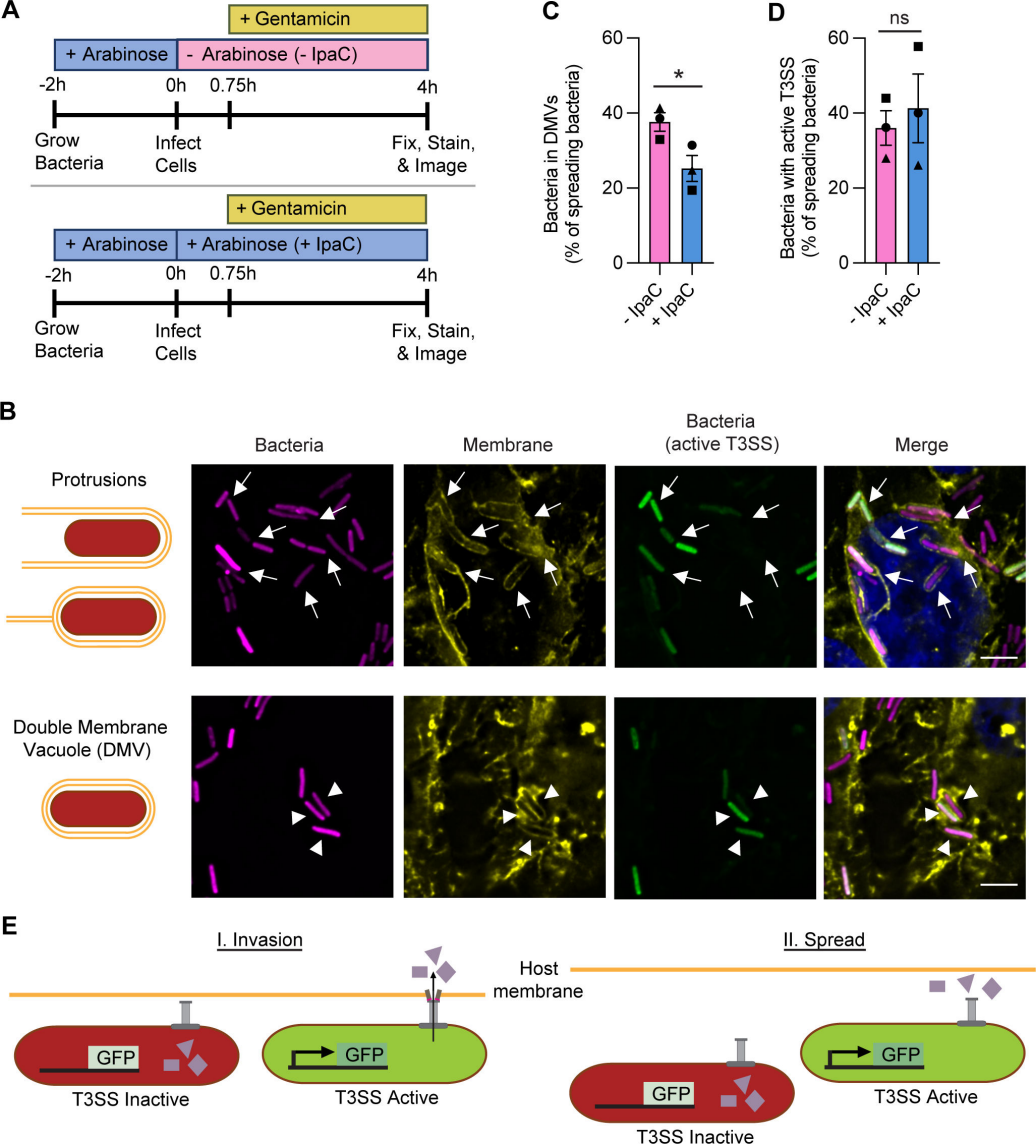
IpaC contributes to bacterial escape from DMVs, but effector secretion is activated independent of IpaC. (**A**) Schematic showing the experimental protocol. + or – IpaC indicates whether arabinose was present in the cell culture media to induce or not induce the production of IpaC throughout infection. Gentamicin was added at 45 minutes of infection to kill extracellular bacteria. (**B**) Schematics depict characteristic appearance of bacteria in protrusions or DMVs. Representative immunofluorescence images of HeLa pmbYFP cells infected with bacteria for 4 hours. Top: bacteria in protrusions; bottom: bacteria in DMVs. Arrows, bacteria in protrusions; arrowheads, bacteria in DMVs; magenta, all bacteria; yellow, HeLa plasma membranes; green, bacteria with active T3SS; blue, DNA; scale bar, 5 µm. (**C and D**) Percentage of bacteria that are spreading (present in DMV or protrusion) that are in DMVs (**C**) or that have active T3SS (**D**) from images represented in (B) Bacteria producing (+IpaC, light blue) or not producing (−IpaC, pink) IpaC during infection. Data are mean ± SEM of three independent experiments; each experiment is matched by symbol. ns (not significant), *P < 0.05 by paired *t*-test. (**E**) Schematic depicting the regulation of T3SS secretion at invasion and spread. Secretion of effectors and transcription of GFP from the TSAR are induced from (I) the formation of a translocation-competent pore in the host membrane at invasion or (II) the contact of the T3SS with the host membrane during spread.

During invasion, T3SS secretion is triggered by the interaction of IpaC with intermediate filaments (8, 9). During spread, intermediate filaments are dispensable, and activation of the secretion of T3SS effectors occurs in their absence and is associated with the interaction of bacteria with the plasma membrane (10, 16). Since IpaC associates with the plasma membrane at spread (Fig. 1), we asked whether T3SS activation during spread is dependent on IpaC. Upon infecting HeLa cells with bacteria that produced or lacked IpaC, we examined T3SS activation during spread (Fig. 2). To enable visualization of bacteria in a protrusion or DMV, we used HeLa cells that produce a membrane-anchored YFP (pmbYFP) (26) (Fig. 2B). As above, these cells were infected with *Sf *∆*ipaC::ipaC* so that IpaC abundance was regulated by the presence of arabinose. These bacteria also encoded a fluorescent reporter of T3SS secretion (pTSAR), which inducibly produces GFP when the T3SS effector OspD1 is secreted and constitutively produces mCherry (16). Here, we used secretion of OspD1 as a general indicator of T3SS activation (8–10, 16, 24). DMVs begin to form around 2 hours of infection (19)(Fig. 1B); we examined infection at 4 hours when spread is robust (Fig. 2B and Fig. S2A) and IpaC produced during invasion is lost (Fig. 1). As anticipated from previous studies (15, 16), the percentage of spreading bacteria present in DMVs increased when bacteria did not produce IpaC (Fig. 2C and Table S1), which confirmed that IpaC is required for efficient escape from DMVs. During invasion, the activation of T3SS secretion requires both pore formation in the plasma membrane and an activation step that is dependent on IpaC interaction with intermediate filaments (9, 18). By contrast, during spread, T3SS activation is associated with membrane contact (16). We observed T3SS activation was efficient for bacteria that were spreading at 4 hours of infection, even for bacteria that lacked IpaC and were unable to form pores (Fig. 2B through D and Table S1). We generally observed that more bacteria produced GFP in the absence of IpaC (Fig. S2B and C and Table S1), suggesting that bacteria contacting host membrane, such as when trapped in the DMV, maintain active T3SS longer than bacteria producing IpaC that quickly escape the DMV and spread. Regardless of the reason for bacteria lacking IpaC to produce more GFP, these data show that during spread, T3SS activation and effector secretion occur in the absence of IpaC (Fig. 2E). They further show that during spread, the regulation of T3SS activation and effector secretion is distinct from that at invasion. Altogether, these data show that IpaC is required for *S. flexneri* to escape the DMV but is dispensable for activating T3SS secretion during spread.

### IpaC is required in both membranes of the DMV for efficient bacterial escape

**Fig 3:**
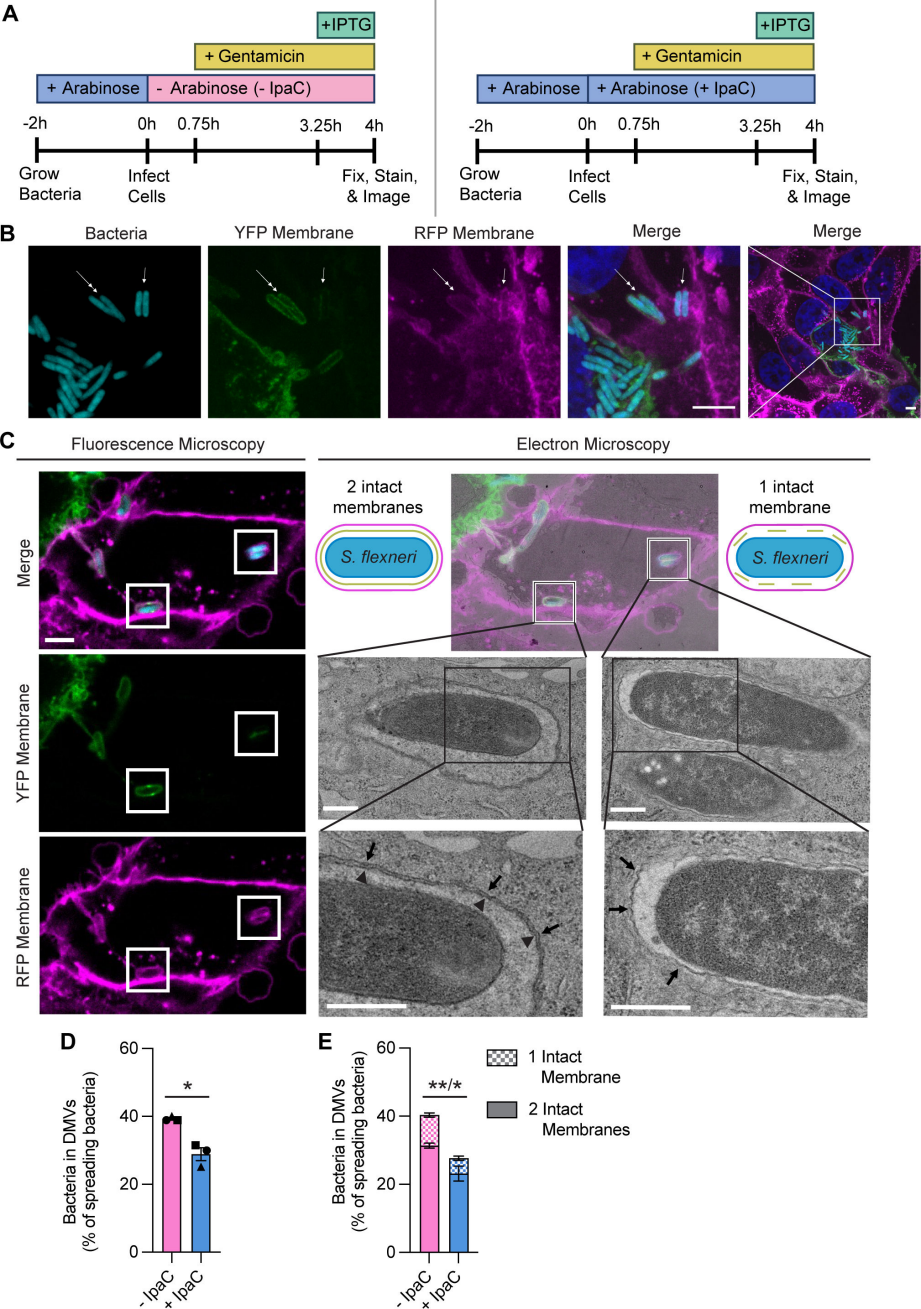
IpaC is required in both membranes of the DMV for efficient bacterial escape. (A) Schematic showing the experimental protocol. + or – IpaC indicates whether arabinose was present in the cell culture media to induce or not induce the production of IpaC throughout infection. Gentamicin was added at 45 minutes of infection to kill extracellular bacteria. IPTG was added 45 minutes prior to fixation to induce production of CFP. (B) Representative immunofluorescence images of HeLa pmbYFP and HeLa pmbRFP cells infected with bacteria for 4 hours. Double-headed arrows: bacteria in a DMV with two intact membranes (both donor and recipient cell membranes); single-headed arrows: bacteria in a DMV with one intact membrane (recipient cell membrane only). White rectangle: area of infection that originates from a cell with a different membrane fluorescence than surrounding cells. Cyan, bacteria; green, HeLa pmbYFP plasma membranes; magenta, HeLa pmbRFP plasma membranes; blue, DNA; scale bars, 5 µm. (C) Representative fluorescence images (left) of the same bacteria imaged by electron microscopy (right) using CLEM to identify positions with bacteria in one or two intact membranes from three independent experiments. 12 total positions were imaged, accounting for 19 bacteria. Cyan, bacteria; green, HeLa pmbYFP plasma membranes; magenta, HeLa pmbRFP plasma membranes. Fluorescence microscopy: white boxes, bacteria in two (left) or one (right) membrane; scale bar, 5 µm. Electron microscopy: black boxes, areas of in-focus DMV membrane(s); arrows, recipient membrane; arrowheads, donor membrane; scale bar, 0.5 µm. (D and E) From images represented in panel B, percentage of bacteria that are spreading (present in a DMV or protrusion) that are in DMVs (D) and percentage of bacteria in DMVs that have two intact membranes (solid) or one intact membrane (checkered) (E). Bacteria producing (+IpaC, aqua) or not producing (−IpaC, magenta) IpaC during infection. Data are mean ± SEM of three independent experiments. (D) Each experiment is matched by symbol. *P < 0.05 by paired *t*-test. (E) Comparison of the proportion of bacteria in DMVs with two intact membranes/one intact membrane. *P < 0.05, **P < 0.01 by two-way ANOVA with Holm-Sidak’s multiple comparisons test.

At invasion, the escape of *S. flexneri* from a vacuole composed of a single membrane requires the T3SS insertion of IpaC into the membrane (27–30). At spread, we found that *S. flexneri* requires IpaC to escape from the DMV (Fig. 2) and that IpaC is associated with the membrane (Fig. 1), but it remained uncertain whether IpaC was required for disruption of one or both membranes of the DMV. To determine which membrane(s) IpaC disrupts, we infected a monolayer consisting of a 1:1 mix of HeLa pmbYFP (26) and HeLa cells producing membrane-anchored RFP (pmbRFP) (31) with *Sf *∆*ipaC::ipaC* that produced CFP upon induction with IPTG (Fig. 3A). By focusing our investigation on DMVs that formed between cells with two distinct membrane-anchored fluorescent proteins, we could discriminate whether the bacterium-containing DMVs contained only the recipient cell membrane or both the donor and recipient cell membranes (Fig. 3B). Using correlative light and electron microscopy (CLEM) (32, 33), in which spinning disc fluorescence microscopy is correlated with transmission electron microscopy (TEM) to provide higher resolution information on events identified with fluorescence, we confirmed that signals from our fluorescence microscopy indicated one or two membranes (Fig. 3C). Similar to Fig. 2C, in this assay, bacteria lacking IpaC more slowly escape from DMVs, leading to a similarly increased percentage of spreading bacteria in DMVs (Fig. 3D and Table S2), indicating that the presence of the two fluorescent markers did not significantly alter the phenotype. When we determined the proportions of DMVs that had one or two intact membranes in each condition, we found that the absence of IpaC increased bacteria within vacuoles containing one or two membranes (Fig. 3E and Table S2). These data indicate that IpaC enhances the disruption of both membranes of the DMV during spread and, by visualizing some bacteria present in vacuoles with a single membrane, suggest there is a sequential breakdown of the membranes of the DMV.

### IpaC is required for efficient disruption of the recipient cell membrane of the DMV

**Fig 4:**
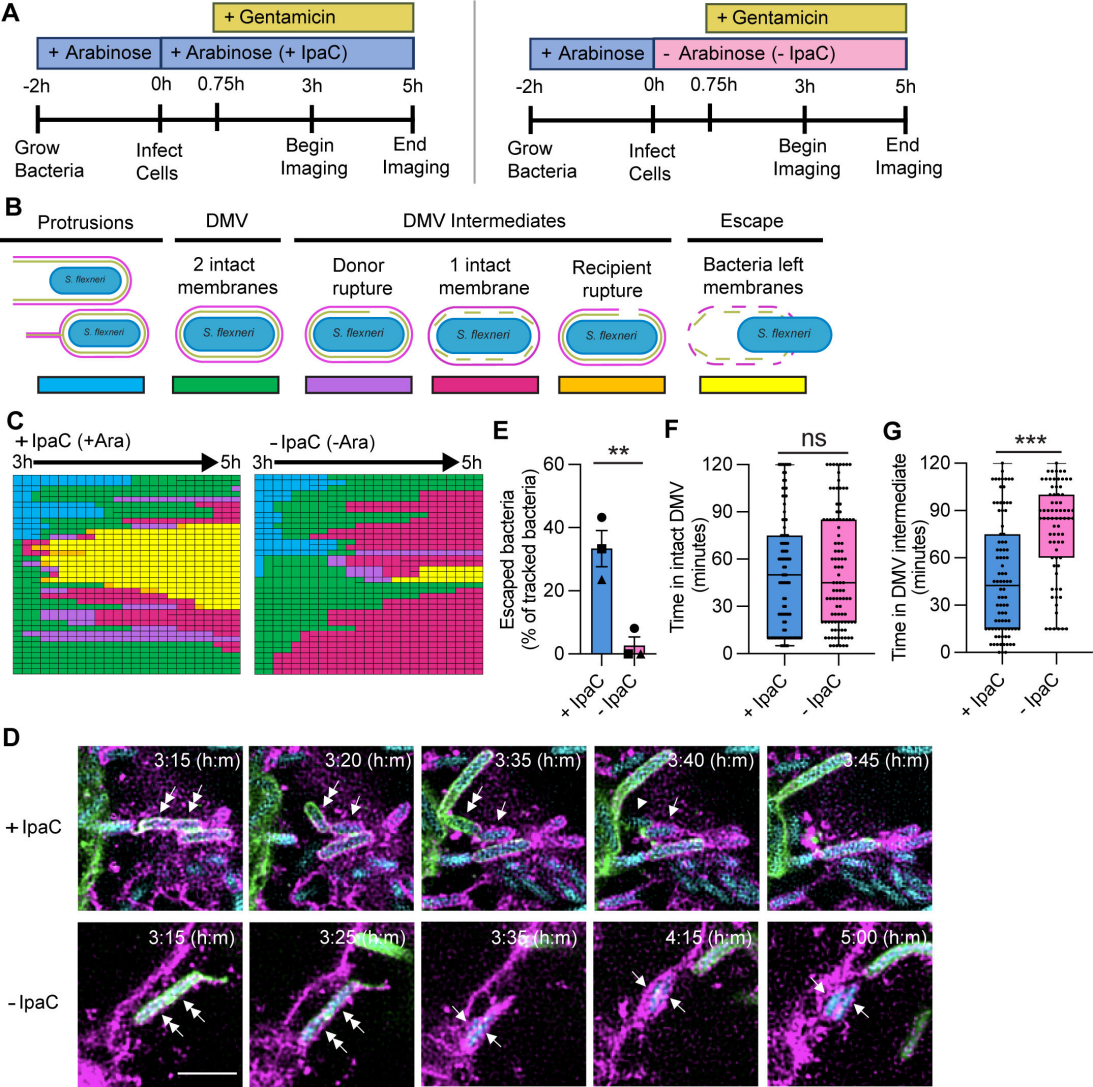
IpaC is required for efficient disruption of the recipient cell membrane of the DMV. (**A**) Schematic showing the experimental protocol. + or – IpaC indicates whether arabinose was present in the cell culture media to induce or not induce the production of IpaC throughout infection. Gentamicin was added at 45 minutes of infection to kill extracellular bacteria. Images were taken every 5 minutes over 2 hours beginning at 3 hours of infection. (**B**) Schematic of the stages of spreading bacteria and their color coding in panel **C**. Representative experiment depicting tracked bacteria. Each box represents one bacterium at one 5 minute interval. Larger boxes represent one bacterium before it divided. Twenty-eight to 37 individual bacteria were tracked per condition per experiment. (**D**) Representative immunofluorescence images of bacteria producing IpaC (top) or lacking IpaC (bottom) from the start of DMV resolution until escape (+ IpaC, top) or not (−- IpaC, bottom). Imaging began at 3 hours of infection, and images were taken every 5 minutes until 5 hours of infection. Cyan, bacteria; green, HeLa pmbYFP plasma membranes; magenta, HeLa pmbRFP plasma membranes; scale bar, 5 µm; double-headed arrows, bacteria in DMVs with two intact membranes; single-headed arrows, bacteria within a DMV with a disrupted donor membrane but intact recipient membrane; arrowheads, bacteria within a DMV with both membranes disrupted; no arrows, bacteria have escaped and regained motility. (**E**) Percentage of tracked bacteria that escaped from DMVs by 5 hours of infection. Data are mean ± SEM of three independent experiments; each experiment is matched by symbol. **P < 0.01 by Student’s *t*-test. (**F and G**) Time bacteria spent within fully intact DMVs (**F**) or DMV intermediates (**G**). Data are median and 25th and 75th percentiles of data set (boxes) of all tracked bacteria in three independent experiments; each dot represents a tracked bacterium. ns, not significant; ***P < 0.001 by Mann-Whitney test.

To understand the kinetics of IpaC disruption of DMV membranes, we performed live microscopy on a monolayer consisting of a 1:1 mix of HeLa pmbYFP and HeLa pmbRFP cells infected with *Sf *∆*ipaC::ipaC* that produced CFP constitutively (Fig. 4A). Beginning at 3 hours of infection, we tracked individual bacteria every 5 minutes starting from protrusions or DMVs until bacteria escaped or 5 hours of infection (Fig. 4A through C; Fig. S3A). We observed that bacteria producing IpaC escaped within the 2-hour period significantly more frequently than bacteria lacking IpaC (Fig. 4C through E and Fig. S3A). Time-lapse imaging enabled identification of several phenotypic processes leading to the disruption of the DMV membranes; these intermediate steps were classified (Fig. 4B) as follows: (i) rupture of the donor cell membrane with an intact recipient cell membrane (Fig. S3B, top), (ii) dissolution of the donor cell membrane and only the recipient cell membrane visible (Fig. 3D), and (iii) rupture of the recipient cell membrane, regardless of whether both membranes remained largely visible (Fig. S3B, bottom). Whereas bacteria lacking IpaC trended toward remaining in a fully intact DMV for longer than bacteria producing IpaC (Fig. 4F), we observed bacteria lacking IpaC remained in at least one of these intermediate steps significantly longer compared to bacteria producing IpaC (median of 85 vs 42.5 minutes) (Fig. 4G). Altogether, these data indicate that IpaC is required for disruption of the recipient cell membrane of the DMV to enable bacterial escape.

### The formation of translocation-competent pores is essential for spread

Mechanoporation and translocon pore formation by the T3SS enables bacterial escape from the vacuole associated with invasion (28, 29). Thus, we speculated that the ability of IpaC to form a pore would be required to lyse the membranes of and to escape from the DMV. To test this, we made use of mutations that disrupt the ability of IpaC to form a functional pore. Loss of the C-terminus disrupts the interactions that enable docking of the T3SS needle to the T3SS pore, the ability to interact with β-catenin, and the translocation of effectors (9–11, 17). Deletion of and mutations around the coiled-coil region of IpaC disrupt the ability of the pore to undergo conformational changes that enable translocation of effectors from *S. flexneri* into the host cytosol (24). Because some mutations of IpaC prevent T3SS-mediated effector translocation required for invasion, we created *S. flexneri* strains that produce an inducible version of wild-type IpaC, which supports invasion and whose abundance becomes undetectable by spread in the absence of continuous induction, using a similar induction approach to that in Fig. 1. We generated IpaC-FLAG, which includes a 1X FLAG tag on the inducible version of IpaC at amino acid position 37 and which is similar in functionality to wild-type IpaC, as it complements *S. flexneri* Δ*ipaC* to form pores and to translocate effector proteins (Fig. S4). In the same strain, a second version of IpaC, which contains mutations that impact pore formation, is constitutively produced, thereby enabling the study of the effect of each of these IpaC mutations on intercellular spread. We differentiate IpaC-FLAG from the constitutive IpaC by a size shift on western blot (Fig. S5A). Plasmids encoding the inducible IpaC-FLAG and constitutive IpaC variants were transformed into *S. flexneri* Δ*ipaC*, and resulting strains were tested for IpaC production and secretion; T3SS secretion remained regulated by Congo red (34) in the presence of IpaC variants and IpaC-FLAG (Fig. S5), which demonstrated that these mutations neither affect the ability of IpaC variants to be secreted nor the activation of the T3SS in general.

**Fig 5:**
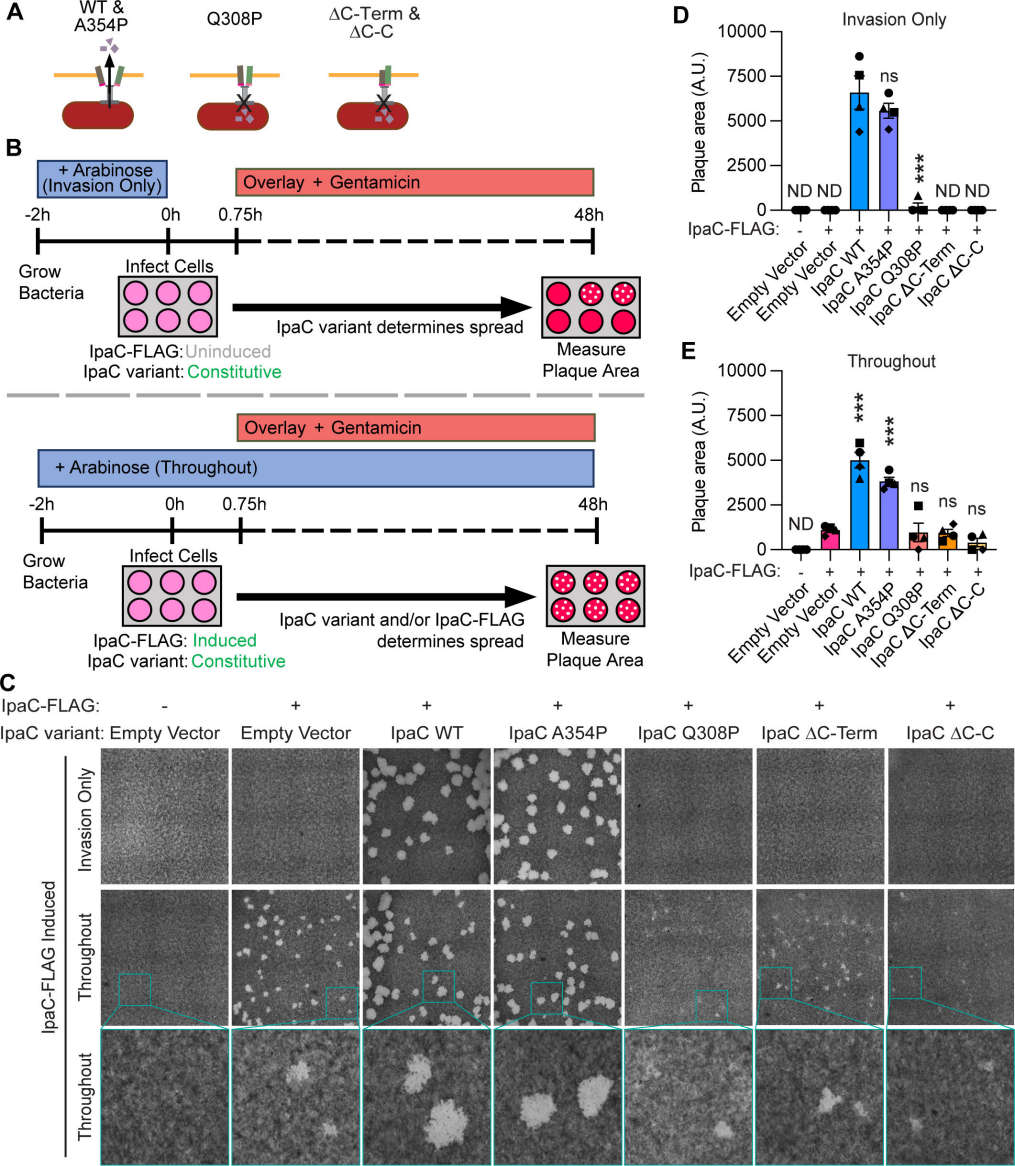
The formation of translocation-competent pores is essential for spread. (**A**) Summary of the translocation-competency of pores formed by IpaC variants at invasion. (**B**) Schematic showing the experimental protocol. “Invasion Only” indicates arabinose was only present in the back dilution to induce the production of IpaC-FLAG to enable invasion. “Throughout” indicates arabinose was present in the back dilution and throughout infection to induce IpaC-FLAG production for both invasion and spread. At 45 minutes of infection, a 1% overlay containing gentamicin was added to kill extracellular bacteria. (**C**) Representative images of plaque assays after 48 hours of infection of MEF cells. Top: IpaC-FLAG induced only before infection to enable invasion. Middle: IpaC-FLAG induced throughout the duration of infection. Bottom: zoomed in from teal square in middle panels. C-Term, C-terminus; C-C, coiled-coil. (**D and E**) Quantification of plaque areas from infections where IpaC-FLAG was induced (**D**) for invasion only or (**E**) throughout the infection from images represented in panel C. Data are mean ± SEM of four independent experiments, each experiment matched by symbol. One-way ANOVA with Dunnett’s multiple comparisons test. ND (no detected plaques), ns (not significant), ***P < 0.001.

Previous investigation of these IpaC variants during invasion showed that they insert in plasma membranes (8, 9, 24) and have varying functionality (Fig. 5A). IpaC Q308P forms pores with smaller openings that lyse membranes less efficiently than WT IpaC (24). IpaC Δcoiled-coil is unable to lyse membranes (24). Neither IpaC Q308P nor IpaC Δcoiled-coil translocates effectors during invasion (24). IpaC A354P translocates effectors during invasion, but with reduced efficiency compared to WT IpaC (24). We characterized the ability of these IpaC variants to support intercellular spread using a plaque assay (Fig. 5B). In this assay, the number of plaques correlates with the efficiency of invasion, and the area of plaques is associated with the efficiency of intercellular spread. We induced production of IpaC-FLAG prior to invasion so that strains could invade cells regardless of which constitutive version of IpaC was produced; induction of IpaC-FLAG was stopped at the time of invasion so that the impact of IpaC mutations on intercellular spread could be observed (Fig. 5B, top). Indeed, IpaB and constitutively produced IpaC variants were present in the plasma membrane throughout the experiment, but IpaC-FLAG produced from induction prior to invasion was undetectable at 4 hours (Fig. S6). Plaques were not detected from strains lacking IpaC nor from strains producing IpaC Q308P, IpaC ΔC-terminus, or IpaC Δcoiled-coil; in contrast, large plaques were formed by strains producing WT IpaC or IpaC A354P, which support translocation (Fig. 5C and D). When production of IpaC-FLAG, which also supports translocation, is maintained throughout the duration of the assay (Fig. 5B, bottom), all strains producing IpaC-FLAG formed plaques (Fig. 5C and E); IpaC-FLAG did not rescue plaques to the full size of those produced by plaque-forming derivatives (IpaC WT and IpaC A354P), likely due to the lower levels of IpaC-FLAG compared to these variants (Fig. S5). Together, these data show that defects in plaque formation observed for particular IpaC variants were not the result of the strain being unable to invade cells or insert into the membrane during spread, but rather, due to the inability of specific constitutively produced IpaC mutants to support intercellular spread. Because in the absence of translocation-competent IpaC-FLAG during spread, plaques were observed only for strains producing variants of IpaC that could both form pores and translocate effectors, these findings indicate that spread requires pores that are sufficiently open so that effectors can translocate through them. Overall, these data suggest that IpaC is required during spread for the formation of a translocation-competent pore.

### Translocation-competent pores are required for *S. flexneri* **t**o recruit Rac1 and to escape from DMVs

The enzymatic activity of the T3SS effectors, IcsB and IpgB1, contributes to bacterial escape from the DMV (26, 35–42). However, how effectors gain access across the target membranes of the DMV is unclear. It is presumed there may be translocation, but it is unclear whether the T3SS translocon pore functions to translocate effectors, for several reasons: (i) the DMV is composed of two plasma membranes, and it is unknown whether or how the pore might mediate effector translocation across both donor and recipient cell membranes; (ii) the regulation of T3SS-mediated secretion is uncoupled from pore formation (Fig. 2), which suggests translocation may not involve the IpaB-IpaC translocon pore or may not occur.

We sought to investigate the relationship between effector function in the recipient cell and the ability to form translocation-competent translocon pores. IpgB1 regulates Rho family GTPases and recruits Rac1 around DMVs in a manner that enables escape from DMVs (35, 40–42); first, we confirmed that efficient recruitment of Rac1 to DMVs is dependent on the expression of *ipgB1* in our system (Fig. S7A). Similar to previous investigations (35), we infected HT-29 pmbYFP intestinal epithelial cells with WT *S. flexneri*, *S. flexneri* Δ*icsB*, or *S. flexneri* Δ*ipgB1*, and at 5 hours of infection, we observed Rac1 was recruited to fewer DMVs in cells infected with *S. flexneri* Δ*ipgB1* (Fig. S7B through D and Table S3). This was not simply due to the amount of time spent in the DMV; rather, it was specific to the absence of IpgB1. *S. flexneri* Δ*icsB* is delayed in escape from DMVs, similar to *S. flexneri* Δ*ipgB1*, but *S. flexneri* Δ*icsB* efficiently recruits Rac1 to DMVs (Fig. S7C through E and Table S3). We additionally confirmed these observations in HeLa pmbYFP cells (Fig. S8 and Table S4). Thus, Rac1 recruitment requires IpgB1, and Rac1 recruitment is not diminished simply due to slower DMV escape.

**Fig 6:**
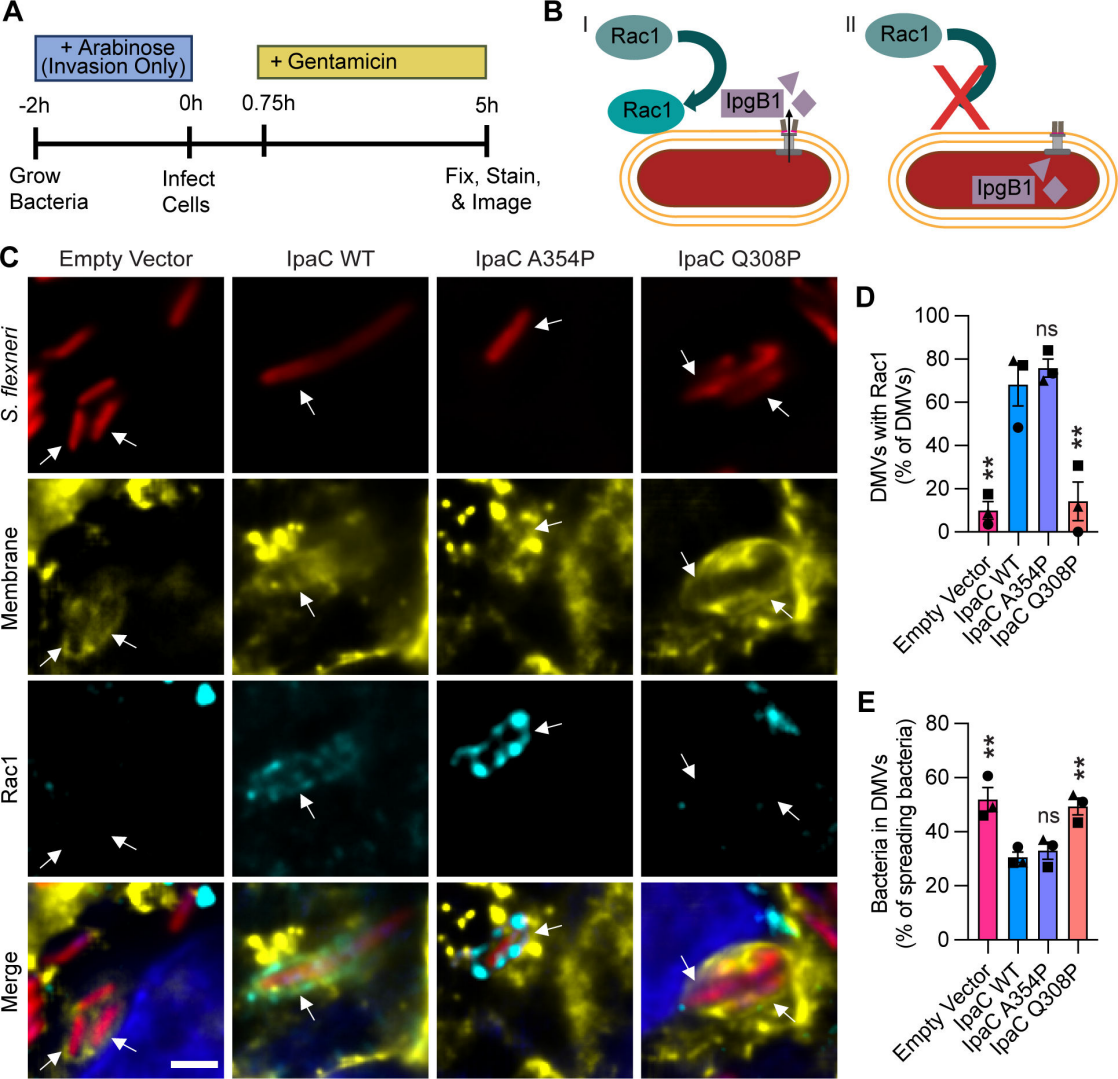
Translocation-competent pores are required for *S. flexneri* to recruit Rac1 and to escape from DMVs. (**A**) Schematic showing the experimental protocol. “Invasion Only” indicates arabinose was only present in the back dilution to induce the production of IpaC-FLAG to enable invasion. Gentamicin was added at 45 minutes of infection to kill extracellular bacteria. (**B**) Schematic. (I) A translocation-competent pore enables recruitment of Rac1 to a DMV by the translocated effector IpgB1. (II) A translocation-incompetent pore is unable to facilitate Rac1 recruitment to a DMV since effectors are not translocated. (**C**) Representative immunofluorescence images of HT-29 pmbYFP cells infected with bacteria producing indicated IpaC variant at 5 hours of infection. All strains included IpaC-FLAG and were induced only before infection to enable invasion. Bacteria within a DMV (white arrow). Red, bacteria; yellow, HT-29 cell membranes; cyan, Rac1; blue, DNA; scale bar, 2 µm. (**D and E**) From images represented in panel C, percentage of bacteria that are spreading and in DMVs (**D**) and percentage of bacteria within DMVs that colocalize with Rac1 (**E**). Data are mean ± SEM of three independent experiments; each experiment is matched by symbol. ns (not significant), **P < 0.01 by one-way ANOVA with Dunnett’s multiple comparisons test.

We next tested whether the formation of translocation-competent pores was associated with recruitment of Rac1 to and bacterial escape from DMVs. We infected HT-29 pmbYFP cells with *S. flexneri* Δ*ipaC* producing an inducible IpaC-FLAG and an empty vector or constitutive version of IpaC (WT IpaC, IpaC A354P, or IpaC Q308P). We induced the production of IpaC-FLAG only during the back dilution so that all strains could invade cells (Fig. 6A). The percentage of DMVs associated with Rac1 and bacteria within DMVs was quantified by fluorescence microscopy. Whereas all strains express *ipgB1* and thus have the capacity to secrete IpgB1 to recruit Rac1, we observed that bacteria producing IpaC Q308P (forms a pore that is too narrow to translocate effectors) (24) and bacteria that harbor an empty vector (are unable to form a pore and do not translocate effectors) showed less recruitment of Rac1 around DMVs than bacteria producing IpaC WT or IpaC A354P (forms translocation-competent pores) (Fig. 6B through D and Table S5). This shows that mutations in IpaC that prevent translocation also block Rac1 recruitment. In addition, bacteria with an empty vector or producing IpaC Q308P were more likely to be present in DMVs than bacteria producing WT IpaC or IpaC A354P (Fig. 6C through E and Table S5). Similar observations were made in HeLa pmbYFP cells infected with these strains (Fig. S9 and Table S6). Overall, these data show that a translocation-competent pore is required for activity of effectors that promote escape from DMVs, strongly implying that IpaC-dependent effector translocation occurs during spread.

### Translocation-competent pores are required for disruption of both membranes of the DMV

**Fig 7:**
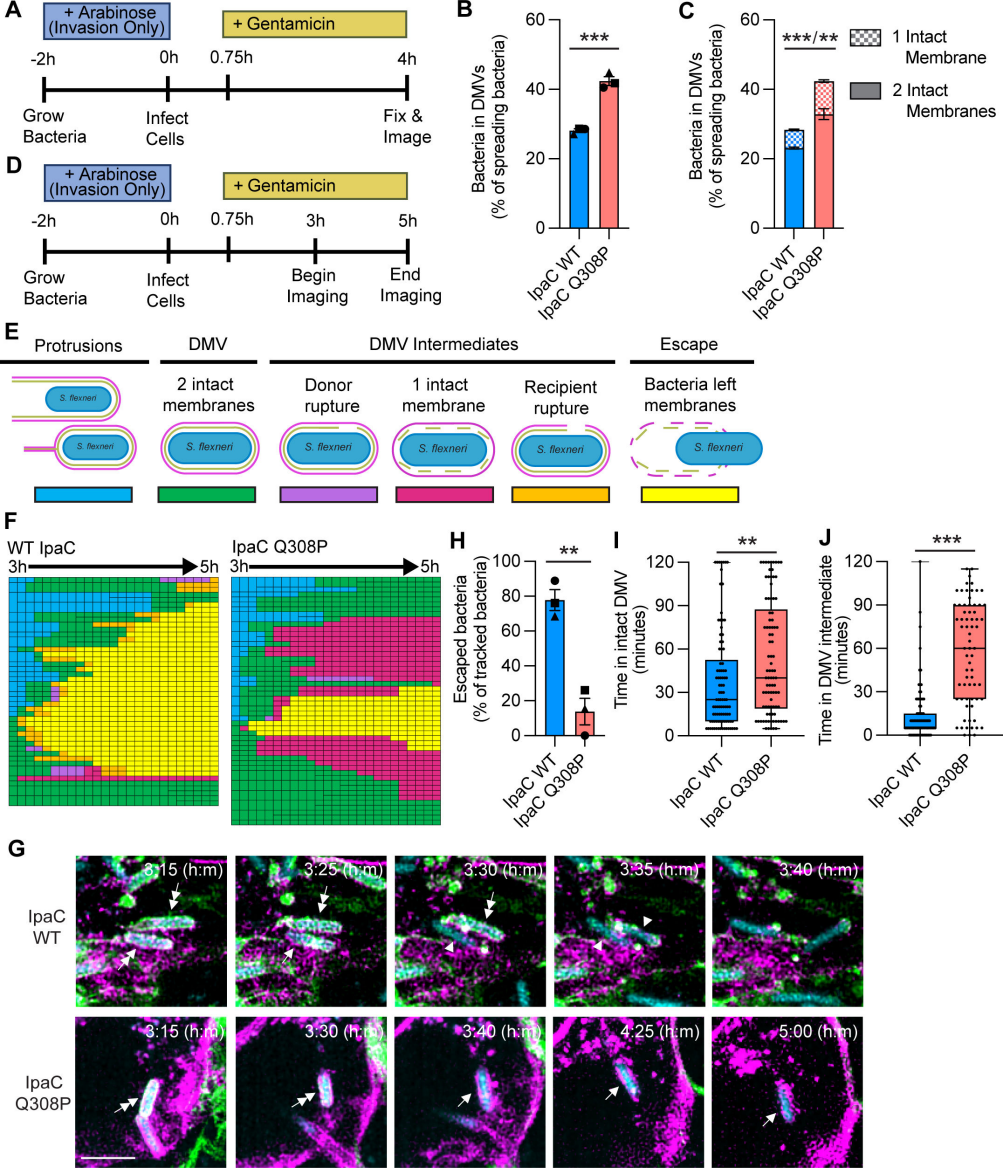
Translocation-competent pores are required for disruption of both membranes of the DMV. (**A**) Schematic showing the experimental protocol. “Invasion Only” indicates arabinose was only present in the back dilution to induce the production of IpaC-FLAG to enable invasion. Gentamicin was added at 45 minutes of infection to kill extracellular bacteria. (**B**) Percent of bacteria that are spreading and in DMVs. (**C**) Percentage of bacteria in DMVs that have two intact membranes (solid) or one intact membrane (checkered). (**B and C**) Data are mean ± SEM of three independent experiments. (**B**) Each experiment is matched by symbol. ***P < 0.001 by Student’s *t*-test. (**C**) Comparison of the proportion of bacteria in DMVs with two intact membranes/one intact membrane. **P < 0.01, ***P < 0.001, by two-way ANOVA with Holm-Sidak’s multiple comparisons test. (**D**) Schematic showing the experimental protocol. Invasion Only indicates arabinose was only present in the back dilution to induce the production of IpaC-FLAG to enable invasion. Gentamicin was added at 45 minutes of infection to kill extracellular bacteria. Images were taken every 5 minutes over 2 hours beginning at 3 hours of infection. (**E**) Schematic of the stages of spreading bacteria and their color coding in panel **F**. Representative experiment depicting tracked bacteria. Each box represents one bacterium at one 5 minute interval. Larger boxes represent one bacterium before it divided. Fifteen to 50 individual bacteria ( were tracked per IpaC variant per experiment. (**G**) Representative time-lapse immunofluorescence images of bacteria producing IpaC WT (top) or IpaC Q308P (bottom) from the start of DMV resolution until escape (IpaC WT, top) or not (IpaC Q308P, bottom). Imaging began at 3 hours of infection, and images were taken every 5 minutes until 5 hours of infection. Cyan, bacteria; green, HeLa pmbYFP plasma membranes; magenta, HeLa pmbRFP plasma membranes; scale bar, 5 µm; double-headed arrows, bacteria in DMVs with two intact membranes; single-headed arrows, bacteria within a DMV with a disrupted donor membrane but intact recipient membrane; arrowheads, bacteria within a DMV with both membranes disrupted; no arrows, bacteria have escaped and regained motility. (**H**) Percentage of tracked bacteria that escaped from DMVs by 5 hours of infection. Data are mean ± SEM of three independent experiments; each experiment is matched by symbol. **P < 0.01 by Student’s *t*-test. (**I and J**) Time bacteria spent within fully intact DMVs (**I**) or DMV intermediates (**J**). Data are median and 25th and 75th percentiles of data set (boxes) of all tracked bacteria in three independent experiments (median and 25th percentile are the same in IpaC WT in panel **J**; each dot represents a tracked bacterium. **P < 0.01, ***P < 0.001 by Mann-Whitney test.

We sought to determine whether our finding that the absence of IpaC increased bacteria in DMVs with either one or two intact membranes (Fig. 3) was due to the lack of translocation-competent pores. Similar to infections described above, we infected a monolayer consisting of a 1:1 mix of HeLa pmbYFP (26) and HeLa pmbRFP (31) cells with bacteria producing either IpaC Q308P (forms a pore that is too narrow to translocate effectors) or WT IpaC (forms a pore and translocates effectors) (Fig. 7A). Bacteria within DMVs with only the recipient cell membrane intact or with both donor and recipient cell membranes intact were quantified, as in Fig. 3. We observed more bacteria within DMVs when bacteria produced IpaC Q308P (Fig. 7B and Table S7), similar to when no IpaC was produced (Fig. 3). We also observed an increase in bacteria within DMVs with one or two intact membranes (Fig. 7C and Table S7), indicating that translocation-competent pores are required for efficient disruption of both membranes of the DMV.

To understand how a translocation-competent pore contributes to the kinetics of disrupting the membranes of the DMV, we performed live microscopy and tracked individual bacteria, as described in Fig. 4, with a monolayer consisting of a 1:1 mix of HeLa pmbYFP and HeLa pmbRFP cells infected with bacteria producing either WT IpaC or IpaC Q308P (Fig. 7D through G and Fig. S10). We observed that bacteria producing WT IpaC escaped within the 2-hour period significantly more frequently than bacteria producing IpaC Q308P (Fig. 7F through H and Fig. S10). Bacteria that produced IpaC Q308P not only remained in a fully intact DMV significantly longer than bacteria producing WT IpaC (median of 40 vs 15 minutes) (Fig. 7I) but also remained in an intermediate step significantly longer than bacteria producing WT IpaC (median of 60 vs 5 minutes) (Fig. 7J). Altogether, these data indicate that translocation-competent pores are required to disrupt both membranes of the DMV to enable bacterial escape into the recipient cell cytosol, thereby facilitating bacterial spread.

## DISCUSSION

Intercellular spread is essential for the virulence associated with *S. flexneri* infections, and IpaC is essential for intercellular spread. Here, we built upon previous investigations of IpaC function during *S. flexneri* invasion of epithelial cells to characterize IpaC function during intercellular spread, with a specific focus on the role of IpaC in escape from DMVs. We show new evidence that T3SS pore proteins are inserted into host plasma membranes during spread and that translocon-competent pores enable effector function in the recipient cell and efficient disruption of both membranes of the DMV, which leads to efficient escape of *S. flexneri* from the DMV and release of *S. flexneri* into the recipient cell cytosol.

Previous studies show that the T3SS and insertion of IpaC and IpaB are sufficient to lyse the entry vacuole at invasion (27–29), suggesting that the ability to form pores at spread may be required for escape from the DMV. Translocon pores that contain IpaC Q308P lyse the membranes of red blood cells, albeit less efficiently than translocon pores that contain WT IpaC, and are unable to support effector translocation at invasion (24), showing that IpaC Q308P supports formation of a partially open pore. Similar to bacteria not producing IpaC during spread, *S. flexneri* producing IpaC Q308P did not recruit Rac1 to DMVs (Fig. 6D and Fig. S9) and failed to escape from DMVs (Fig. 4, 6E and 7). These findings indicate that formation of a partially open pore is insufficient for Rac1 recruitment, and that effector translocation is required for Rac1 recruitment.

In addition, bacteria that do not form pores or do not translocate effectors showed greater defects in the ability to recruit Rac1 to the DMV than bacteria that were missing just *ipgB1* (Fig. 6D and Fig. S7D). This suggests that in addition to requiring IpgB1, the recruitment of Rac1 around the DMV may depend on the activity of other effectors as well. Overall, these data show that translocon pore formation on its own is not sufficient to mediate bacterial escape from the DMV and that effector translocation is essential.

Unlike invasion, during which bacteria escape from the single membrane of the entry vacuole, during spread, bacteria must escape from both the donor cell membrane and the recipient cell membrane of the DMV. Although we showed that a translocation-competent pore is essential for escape from the DMV, bacteria that could not form pores or formed pores unable to translocate effectors were still able to break down the donor cell membrane (Fig. 3E, 4, and 7).

Interestingly, bacteria producing constitutive WT IpaC were the most efficient at disrupting the donor cell membrane, recipient cell membrane, and escaping, even compared to bacteria that produced WT IpaC when induced by arabinose (Fig. 4C through G and 7F through J). This difference observed in our strains producing WT IpaC is likely because constitutive IpaC is produced from a plasmid with a ColE1 origin that is maintained in bacteria at a higher copy number than IpaC produced from induction of the pBAD promoter on a plasmid with a p15 origin; this also explains the difference in the production of IpaC-FLAG and IpaC variants within the same strain (Fig. S5) and why inducing production of IpaC-FLAG throughout infection resulted in smaller plaque areas than observed in strains producing WT IpaC or IpaC-A354P constitutively (Fig. 5C through E). Together, these data indicate that both abundance and ability of IpaC to form a translocation-competent pore contribute to efficient disruption of the donor cell membrane, and translocation competency is essential to disruption of the recipient cell membrane.

Since effectors are secreted even in the absence of pore formation during spread (Fig. 2D through E), and the donor cell membrane is disrupted regardless of whether a translocation-competent pore is formed (Fig. 3E, 4, and 7), it is possible that effectors assist in disrupting the donor cell membrane. The function of the translocon pore proteins in the breakdown of the donor cell membrane remains uncertain. IpaC releases tension at the membrane, enabling efficient protrusion formation (10), which indicates IpaC is likely present within the donor cell membrane. It is likely IpaC interacts with IpaB to form a pore at this step since a translocation-competent pore enables more efficient donor cell membrane breakdown (Fig. 7I). Whether the breakdown of the donor cell membrane is due to translocation of effectors or mechanical disruption of the membrane from a larger pore is unclear; resolution of this process would require the application of more sophisticated techniques than are currently available.

**Fig 8:**
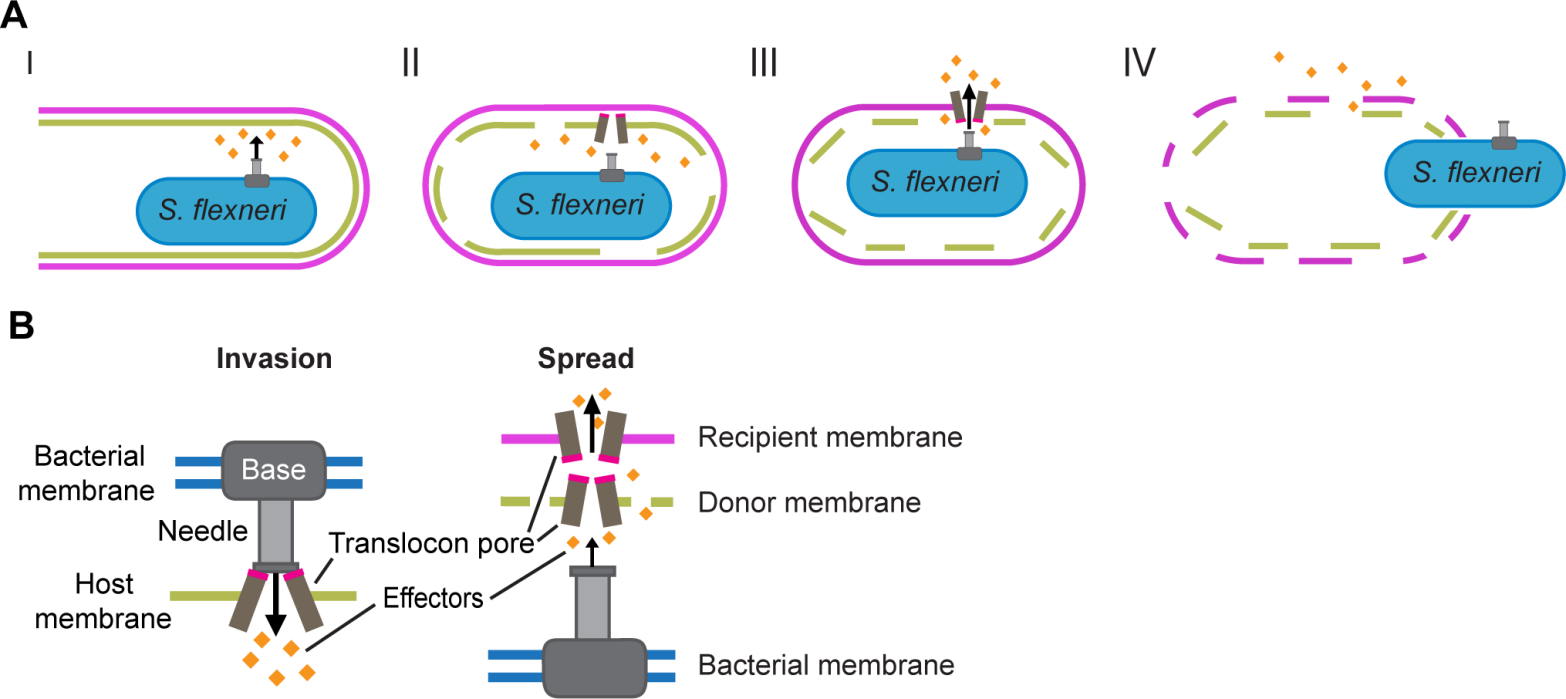
Model of DMV escape and translocation during *S. flexn*eri infection. (**A**) Model of how *S. flexneri* escapes from the DMV. Gold diamonds represent effectors. (I) Upon contact with the host membrane, *S. flexneri* secretes effectors and forms a protrusion. (II) Effectors and insertion of the pore enable disruption of the donor membrane of the DMV. (III) A translocation-competent pore is inserted into the recipient membrane, and effectors are translocated. (IV) Translocated effectors function in the cytosol of the recipient cell to enable bacterial escape from the DMV. (**B**) Schematic of pore insertion and translocation at invasion (left) and speculative model for pore insertion at spread (right). At spread, we hypothesize regions of the pore that interact with the T3SS needle (pink) are oriented away from the T3SS when within the donor membrane, contributing to the differential regulation of the T3SS during spread.

Our data show that a pore capable of translocating effectors is necessary for intercellular spread and disruption of the recipient cell membrane. How translocation might occur in light of the regulatory differences observed in T3SS activity at spread and at invasion is unclear. It is known that during invasion, T3SS-mediated translocation occurs when the effector proteins are loaded into the T3SS base in the bacterial cytosol and then transported through the T3SS needle and across the T3SS translocon pore in a single step. This is facilitated by a close interaction, known as docking (9, 43), of the T3SS needle onto the pore, which results from interactions of the translocon pore protein IpaC with intermediate filaments in the host cell (8). Since intermediate filaments are dispensable for *S. flexneri* during spread (10), the T3SS needle may not need to dock, in which case, translocation may not occur as a single step at spread. In support of this, a mutant that is unable to support docking, IpaC R326W, efficiently activates secretion (10), and we found that IpaC is not required for activation of secretion at spread (Fig. 2D). There is evidence that *Yersinia* T3SS effector translocation can occur in the absence of docking by using a two-step process in which effectors are first secreted through the T3SS needle into the space outside the bacterium, and in a second step, effectors are transported through the T3SS translocon pore into the host cytosol (44). It is uncertain whether contact with the recipient plasma membrane would trigger one- or two-step translocation. The orientation of the recipient cell membrane relative to the bacterium is similar to that which occurs during invasion, so the pore may be inserted in a manner that it is compatible with docking to the T3SS needle, which would enable one-step translocation; alternatively, due to the observed absence of docking and the observed secretion of effectors in the absence of pore formation, we propose that two-step translocation may occur, enabling effectors secreted near the recipient membrane to translocate across the pore and into the recipient host cell cytosol to function in bacterial escape from the DMV during spread (Fig. 8).

During the invasion, the *S. flexneri* T3SS initially interacts with lipids in the outer leaflet of the mammalian membrane, but during spread, *S. flexneri* is in the cell cytosol, such that the T3SS initially interacts with the inner leaflet of the membrane. Both leaflets contain cholesterol, but the outer leaflet maintains a net neutral charge and the inner leaflet has a net negative charge (45, 46). Our data indicate that the regulation of effector secretion at spread is distinct from invasion. We thus speculate that the negative charge of the inner leaflet lipids may trigger secretion of effectors from *S. flexneri* and that proximity of the T3SS needle to the membrane allows the pore proteins IpaC and IpaB to insert without diffusing far from the target membrane. This may be supported by our observation that the IpaC ΔC-terminus and IpaC Δcoiled-coil variants, when secreted, are more abundant in the host membrane during spread than in the supernatant during Congo red induction (Fig. S5 and S6). Although Congo red induces secretion and is also negatively charged (34), these IpaC variants may degrade faster when not stabilized within a membrane soon after secretion. Since both membranes of the DMV are near the T3SS, pore proteins would not have far to diffuse after secretion before inserting into a membrane. These findings are consistent with our model of two-step translocation during spread (Fig. 8).

Interestingly, in our fixed images, we found that a large majority of DMVs had two intact membranes around the entrapped bacteria regardless of whether bacteria produced translocation-competent pores or not (Fig. 3E and 7C). In live imaging of our IpaC variants, when bacteria produced WT IpaC, we still observed a majority of DMVs with two intact membranes, but when bacteria produced IpaC Q308P, we saw a more equal distribution of bacteria in DMVs with either one or two intact membranes, suggesting a larger defect in disrupting the recipient cell membrane than was observed in our fixed images (Fig. 7C and F). Whereas the analysis of fixed images assessed the prevalence of spreading events at one snapshot in time, the analysis for live imaging tracked the fate of bacteria within DMVs or protrusions over time. The two approaches are anticipated to result in slightly different outcomes as the DMVs that resolve are observed in live microscopy, whereas only the DMVs with one or two intact membranes were quantified by fixed microscopy. Thus, these two methods together provide a more comprehensive understanding of the dynamics of DMV escape.

Overall, our results show that the escape of *S. flexneri* from DMVs requires translocation-competent pores and the translocation of effectors. These two processes together lead to disruption of both membranes of the DMV. Since intercellular spread is essential for *S. flexneri* pathogenesis, it is important to understand the distinct regulation and function of the *S. flexneri* T3SS and associated pore proteins during infection. These studies provide insights into how *S. flexneri* regulates the placement of effectors to carry out functional activities both around the bacteria and across membranes.

## MATERIALS AND METHODS

### Bacterial strains and culture

All *Shigella* strains used in this study are isogenic derivatives of *Shigella flexneri* strain 2457T (Table 1). *S. flexneri* strains were cultured in trypticase soy broth with appropriate antibiotics at 37°C at 250 rpm. *ipaC* or *ipaC-FLAG37* was cloned under control of the pBAD promoter on the pBAD33 plasmid. An *ipaC* variant (WT, A354P, Q308P, ΔC-terminus, or Δcoiled-coil) was cloned into a plasmid under the control of a constitutively active promoter; this plasmid also included mCherry under control of the *rpsM* promoter. We named this plasmid pMEIC (mCherry expressing, *ipaC* constitutive). When mCherry was replaced with ECFP, we named this plasmid pCEIC (ECFP expressing, *ipaC* constitutive). Cloning and plasmid preparation were performed using *E. coli* DH10B, and plasmids were selected with appropriate antibiotics in Luria Broth. Plasmids were transformed into *S. flexneri *∆*ipaC* and selected with appropriate antibiotics. Inducible production of IpaC or IpaC-FLAG was enabled by the regulation of IpaC from the pBAD promoter. Constitutive production of IpaC variants was enabled by the *ipaH7.8* promoter.

**TABLE 1:** List of strains used in this study^*a*^

Bacterial strain	Plasmid 1	Plasmid 2	Plasmid 3	Source	Reference
*S. flexneri* 2457T Δ*ipaC*	N/A	N/A	N/A	Laboratory stock	(9)
*S. flexneri* 2457T Δ*ipaC*	pBAD33-ipaC	N/A	N/A	Laboratory stock	(9)
*S. flexneri* 2457T Δ*ipaC*	pBAD33-ipaC-FLAG37	N/A	N/A	This study	This study
*S. flexneri* 2457T Δ*ipaC*	pBAD33-ipaC	pBR322-Afa-1	N/A	Laboratory stock	(18)
*S. flexneri* 2457T Δ*ipaC*	pTSAR	N/A	N/A	Laboratory stock	(9)
*S. flexneri* 2457T ΔipaC	pBAD33-ipaC	pTSAR	N/A	Laboratory stock	(9)
*S. flexneri* 2457T Δ*ipaC*	pBAD33-ipaC	pROEX-Aqua	N/A	Laboratory stock	(10)
*S. flexneri* 2457T Δ*ipaC*	pBAD33-ipaC	pCEIC	N/A	This study	This study
*S. flexneri* 2457T Δ*ipaC*	pBAD33-ipaC-FLAG37	pTSAR	N/A	This study	This study
*S. flexneri* 2457T Δ*ipaC*	pBAD33	pMEIC	N/A	This study	This study
*S. flexneri* 2457T Δ*ipaC*	pBAD33-ipaC-FLAG37	pMEIC	N/A	This study	This study
*S. flexneri* 2457T Δ*ipaC*	pBAD33-ipaC-FLAG37	pMEIC-ipaC	N/A	This study	This study
*S. flexneri* 2457T Δ*ipaC*	pBAD33-ipaC-FLAG37	pMEIC-ipaC-A354P	N/A	This study	This study
*S. flexneri* 2457T Δ*ipaC*	pBAD33-ipaC-FLAG37	pMEIC-ipaC-Q308P	N/A	This study	This study
*S. flexneri* 2457T Δ*ipaC*	pBAD33-ipaC-FLAG37	pMEIC-ipaC-ΔC-terminus	N/A	This study	This study
*S. flexneri* 2457T Δ*ipaC*	pBAD33-ipaC-FLAG37	pMEIC-ipaC-Δcoiled-coil	N/A	This study	This study
*S. flexneri* 2457T Δ*ipaC*	pBAD33	pMEIC	pNG162-Afa-1	This study	This study
*S. flexneri* 2457T Δ*ipaC*	pBAD33-ipaC-FLAG37	pMEIC	pNG162-Afa-1	This study	This study
*S. flexneri* 2457T Δ*ipaC*	pBAD33-ipaC-FLAG37	pMEIC-ipaC	pNG162-Afa-1	This study	This study
*S. flexneri* 2457T Δ*ipaC*	pBAD33-ipaC-FLAG37	pMEIC-ipaC-A354P	pNG162-Afa-1	This study	This study
*S. flexneri* 2457T Δ*ipaC*	pBAD33-ipaC-FLAG37	pMEIC-ipaC-Q308P	pNG162-Afa-1	This study	This study
*S. flexneri *2457T	N/A	N/A	N/A	Laboratory stock	(47)
*S. flexneri *2457T Δ*icsB*	N/A	N/A	N/A	Laboratory stock	(48)
*S. flexneri *2457T Δ*ipgB1*	N/A	N/A	N/A	Laboratory stock	(49)
*S. flexneri *2457T	pBAD33	pMEIC	N/A	This study	This study
*S. flexneri *2457T Δ*icsB*	pBAD33	pMEIC	N/A	This study	This study
*S. flexneri *2457T Δ*ipgB1*	pBAD33	pMEIC	N/A	This study	This study
*E. coli* DH10B	N/A	N/A	N/A	Catalog no. 18290015.	Thermo Fisher
*E. coli* DH10B	pBAD33-ipaC-FLAG37	N/A	N/A	This study	This study
*E. coli* DH10B	pMEIC	N/A	N/A	This study	This study
*E. coli* DH10B	pMEIC-ipaC	N/A	N/A	This study	This study
*E. coli* DH10B	pMEIC-ipaC-A354P	N/A	N/A	This study	This study
*E. coli* DH10B	pMEIC-ipaC-Q308P	N/A	N/A	This study	This study
*E. coli* DH10B	pMEIC-ipaC-ΔC-terminus	N/A	N/A	This study	This study
*E. coli* DH10B	pMEIC-ipaC-Δcoiled-coil	N/A	N/A	This study	This study
*E. coli* DH10B	pCEIC	N/A	N/A	This study	This study
*E. coli* DH10B	pCEIC-ipaC	N/A	N/A	This study	This study
*E. coli* DH10B	pCEIC-ipaC-Q308P	N/A	N/A	This study	This study

^*a*^ “N/A” indicates not applicable.

### Cell culture

HeLa, Caco-2, mouse embryonic fibroblast (MEF) cells, HeLa cells stably expressing plasma membrane-targeted yellow fluorescent protein (pmbYFP) (26) (gift from Hervé Agaisse), HeLa cells stably expressing plasma membrane-targeted red fluorescent protein (pmbRFP) (31) (gift from Rebecca Lamason), or HT-29 cells stably expressing pmbYFP (26) (gift from Hervé Agaisse) were grown in Dulbecco’s modified Eagle’s medium (DMEM) supplemented with 10% fetal bovine serum (FBS) and maintained at 37°C in 5% CO_2_. All cell lines are periodically tested for mycoplasma.

### Fractionation of infected cells

HeLa or Caco-2 cells were seeded at 2 × 10^5^ or 4 × 10^5^ cells, respectively, per well in a six-well plate 48 hours prior to infection. Bacteria were cultured at 37°C at 250 rpm overnight, then back diluted with 1.2% arabinose. Two wells of cells per condition were infected with bacteria at a multiplicity of infection (MOI) of 200. To enhance the efficiency of infection, bacteria expressed the *E. coli* adhesion Afa-1 (9, 18, 50). Bacteria were centrifuged onto cells at 800 × *g* for 10 minutes before incubation at 37°C in 5% CO_2_. Arabinose (1.2%) was or was not included in the media throughout infection to control *ipaC* expression from the pBAD promoter during infections with *S. flexneri *∆*ipaC*::pBAD33-*ipaC*. To test IpaC variants at spread, arabinose was only included in the bacterial culture media prior to infections with *Sf *∆*ipaC*::pBAD33-*ipaC*-FLAG37 + pMEIC *ipaC* variant strains. After 45 minutes, cells were washed and remaining extracellular bacteria were killed by the addition of 25 µg/mL gentamicin. Cells were fractionated at the indicated time points using detergents, as done previously (8–10, 18, 23–25). In brief, cells were washed three times with ice-cold 150 mM Tris, pH 7.4, and scraped into 150 mM Tris, pH 7.4, containing protease inhibitors (protease inhibitor cocktail, complete mini-EDTA free; Roche). Scraped cells were collected and pelleted at 3,000 × *g* for 3.5 min at room temp. The pelleted cells were washed once, resuspended in 150 mM Tris, pH 7.4, containing protease inhibitors and 0.2% saponin, and incubated on ice for 20 min. The suspension was centrifuged at 16,900 × *g* for 30 min at 4°C. The supernatant, which contains the cytosol fraction, was transferred to a fresh tube. The pellet was resuspended in 150 mM Tris, pH 7.4, containing protease inhibitors and 0.5% Triton X-100, incubated on ice for 30 min, and centrifuged at 16,900 × *g* for 15 min at 4°C. The supernatant from this spin contained the membrane fraction, and the pellet consisted of the detergent-insoluble fraction, which included intact bacteria. The abundance of IpaC in the membrane and other fractions was determined by western blot.

### Western blot

Samples were run on SDS-PAGE gels and transferred to nitrocellulose using a TurboBlot semi-dry transfer apparatus (BioRad). The following antibodies were used for western blots: rabbit anti-IpaC (51) (diluted 1:10,000, incubated 2 hours at room temperature, gift from Wendy Picking), mouse anti-IpaB (52) (1:15,000, 2 hours at room temperature, gift from Robert Kaminski WRAIR), rabbit anti-GroEL (1:1,000,000, 2 hours at room temperature, catalog no. G6352; Sigma), rabbit anti-caveolin-1 (1:1,000, 2 hours at room temperature, catalog no. C4490; Sigma), mouse anti-E-cadherin (1:500, overnight at 4°C, catalog no. 14-3249-82; Invitrogen), goat anti-rabbit conjugated with horseradish peroxidase (HRP) (1:5,000, 2 hours at room temperature, Jackson ImmunoResearch, catalog no. 115-035-144), goat anti-mouse conjugated with HRP (1:5,000, 2 hours at room temperature, catalog no. 111-035-146; Jackson ImmunoResearch), and goat anti-rat conjugated with HRP (1:5,000, 2 hours at room temperature, catalog no. 115-035-143; Jackson ImmunoResearch). Western blots were developed with Thermo Scientific SuperSignal West Pico or Femto PLUS Chemiluminescent Substrate, and a G:Box (Syngene) was used to acquire the signal. Band intensity was measured using ImageJ (NIH) with the background subtracted.

### Infection and fluorescence staining

HeLa pmbYFP or HT-29 pmbYFP cells were seeded at 2.5 × 10^5^ or 5 × 10^5^ cells, respectively, per well on coverslips in a six-well plate 48 hours prior to infection. For experiments visualizing two membranes of DMVs during infection, HeLa pmbYFP and HeLa pmbRFP cells were mixed 1:1 and seeded at 2.5 × 10^5^ cells per well on coverslips in a six-well plate, or for experiments using live time-lapse microscopy, per chamber in an ibidi two-well chamber slide (catalog no. 80286), 48 hours prior to infection. Bacteria were cultured at 37°C at 250 rpm overnight, then back diluted with 1.2% arabinose. Cells were infected with bacteria at an MOI of 200. Bacteria were centrifuged onto cells at 800 × *g* for 10 minutes before incubation at 37°C in 5% CO_2_. 1.2% arabinose was or was not included in the media throughout infection to control *ipaC* expression from the pBAD promoter during infections with *Sf *∆*ipaC*::pBAD33-*ipaC* + pTSAR, *Sf *∆*ipaC*::pBAD33-*ipaC* + pROEX-Aqua, or *Sf *∆*ipaC*::pBAD33-*ipaC* + pCEIC. To test IpaC variants at spread, arabinose was only included in the bacterial culture media prior to infections with *Sf *∆*ipaC*::pBAD33-*ipaC*-FLAG37 + pMEIC *ipaC* variants or *Sf *∆*ipaC*::pBAD33-*ipaC*-FLAG37 + pCEIC *ipaC* variant strains. After 45 minutes, cells were washed and remaining extracellular bacteria were killed by the addition of 25 µg/mL gentamicin. 10 mM IPTG was added to cells infected with *Sf *∆*ipaC*::pBAD33-*ipaC* + pROEX-Aqua 45 minutes prior to fixation to induce production of ECFP by bacteria. At the indicated timepoints, cells were washed and either imaged live or fixed with 4% paraformaldehyde. If staining for Rac1, fixed cells were permeabilized with 1% Triton X-100 for 15 minutes, then washed and stained with mouse anti-Rac1 (1:50, overnight at 4°C, catalog no. 610650; BD Biosciences) before being washed and incubated with goat anti-mouse Alexa-Fluor 750 (1:100, 2 hours at 25°C, catalog no. A21037; Invitrogen) for 2 hours at 25°C. Cells were washed, stained with Hoechst, and the coverslips were mounted on slides with ProLong Diamond.

### Microscopy of fixed slides

Fluorescence images were acquired on a Nikon Eclipse Ti-2 inverted light microscope equipped with an Orca Fusion BT cMOS camera (Hammamatsu) and Semrock Brightline filters. For experiments visualizing two membranes of DMVs during infection, fluorescence images were acquired on a Nikon Eclipse Ti-2 inverted light microscope equipped with Yokogawa Spinning Disk Field Scanning Confocal System and seven laser line ZIVA light engine. Images were collected with a 100× objective randomly across the coverslip using an automated imaging pipeline created in NIS Elements software (Nikon). Images were deconvolved using a Richardson-Lucy algorithm with 20 iterations. Microscopic images were pseudo-colored and assembled using FIJI (NIH). Z-stacks with 27 slices of 0.20 µm step size were collected per position on a coverslip. To display representative images, slices within a stack were collapsed into a single image based upon the maximum intensity of pixels in each slice using NIS Elements software. NIS Elements software was used to identify GFP-positive and/or mCherry-positive bacteria. Bacteria within DMVs were classified by their complete enclosure within membrane and with no membrane tether as defined previously (26, 35).

### Correlative light and electron microscopy

CLEM was performed as described previously (32). Correlation between spinning disc confocal microscopy, phase contrast, and TEM images was facilitated by the grid pattern, cell distribution, and recognizable cellular landmarks. Z-stack images acquired by spinning disc confocal microscopy were used to accurately relocate the bacteria of interest in serial thin sections. HeLa pmbYFP and HeLa pmbRFP cells were mixed 1:1 and seeded at 2 × 10^5^ cells per dish on gridded dishes (catalog no. P35G-1.5-14-C-GRD; MatTek) 48 hours prior to infection. Bacteria were cultured at 37°C at 250 rpm overnight, then back diluted with 1.2% arabinose. Cells were infected with bacteria at an MOI of 200. Bacteria were centrifuged onto cells at 800 × *g* for 10 minutes before incubation at 37°C in 5% CO_2_. 1.2% arabinose was only included in the bacterial culture media prior to infections with *Sf *∆*ipaC*::pBAD33-*ipaC* + pROEX-Aqua or *Sf *∆*ipaC*::pBAD33-*ipaC*-FLAG37 + pCEIC *ipaC* variant strains. After 45 minutes, cells were washed and remaining extracellular bacteria were killed by the addition of 25 µg/mL gentamicin. 10 mM IPTG was added to cells infected with *Sf *∆*ipaC*::pBAD33-*ipaC* + pROEX-Aqua 45 minutes prior to fixation to induce the production of ECFP by bacteria. At 4 hours of infection, cells were washed, then fixed with 4% paraformaldehyde in 0.1 M sodium cacodylate buffer for 20 minutes before being washed and placed in 0.1 M sodium cacodylate buffer. Images were acquired on a Nikon Eclipse Ti-2 inverted light microscope equipped with Yokogawa Spinning Disk Field Scanning Confocal System and 7-laser line ZIVA light engine. Brightfield images of the entire dish were collected with a 10× objective to capture both the cells and the grid pattern, facilitating later identification of regions of interest in the EM sections. This was followed by brightfield and fluorescence imaging of positions with a 60× objective. Z-stacks with eighteen slices of 0.50 µm step size were collected per position, and 5 × 5 fields of view were stitched together to form composite images. After imaging, cells were fixed in 2.5% (vol/vol) glutaraldehyde in 0.1 M sodium cacodylate buffer at 25°C for 10 minutes before being placed at 4°C overnight. Following five rinses in 0.1 M sodium cacodylate buffer, cells were post-fixed in 1% osmium tetroxide and 0.8% potassium ferricyanide (K₃[Fe(CN)₆]) in 0.1 M sodium cacodylate buffer for 1 hour at room temperature. After rinsing with water, cells were *en bloc* stained with 2% aqueous uranyl acetate overnight. Following five additional water rinses, specimens were dehydrated through a graded ethanol series, infiltrated with Embed-812 resin, and polymerized overnight at 70°C. Regions of interest on resin blocks were identified using previously acquired brightfield images of cells and the dish grid pattern. Selected cells were sectioned using a diamond knife (Diatome) on a Leica EM UC Enuity ultramicrotome and collected onto copper grids, generating 60–70 nm serial sections. Cell distribution patterns were examined at low magnification by TEM to confirm the presence of the cells of interest in the sections. Using z-stack confocal images, the bacteria of interest were further located and imaged. TEM images were acquired using a Tecnai T12 transmission electron microscope (Thermo Fisher) equipped with a LaB₆ filament operating at 80 kV, and captured with an NS15B (15 Mpix) camera (AMT).

### Live time-lapse microscopy

Fluorescence images were acquired at 37°C on a Nikon Eclipse Ti-2 inverted light microscope equipped with Yokogawa Spinning Disk Field Scanning Confocal System and seven laser-line ZIVA light engine. Infected cells were maintained in a humidified chamber at 37°C in 5% CO_2_ while images were collected with a 60× objective at selected points every 5 minutes over 2 hours using an automated imaging pipeline created in NIS elements software (Nikon). Z-stacks with 11 slices of 0.50 µm step size were collected per position. Images were deconvolved using a Richardson-Lucy algorithm with 20 iterations. Microscopic images were pseudo-colored and assembled using FIJI (NIH). To display representative images, slices within a stack were collapsed into a single image based upon the maximum intensity of pixels in each slice using NIS Elements software.

### Plaque assays

MEFs were seeded in six-well plates at 6 × 10^5^ cells per well 24 hours prior to infection. Bacteria were cultured at 37°C at 250 rpm overnight, then back diluted with 1.2% arabinose. Cells were infected with bacteria at an MOI of 0.02 with or without 1.2% arabinose in the cell culture media. Bacteria were centrifuged onto cells at 800 × *g* for 10 minutes and incubated at 37°C in 5% CO_2_ for 1 hour. Media was replaced with DMEM containing 10% FBS, 25 µg/mL gentamicin, 1.2% arabinose where indicated, and 0.5% agarose, and the cells were incubated for 48 hours before an overlay of 0.7% agarose in DMEM with 10% FBS, 25 µg/mL gentamicin, and 0.1% neutral red was added. After 4–6 hours of incubation, plaques were imaged with an ImmunoSpot S6 Universal Visible/Fluorescent Analyzer (Cellular Technology Limited). Plaques were analyzed with a pipeline generated in ImageJ in which images were thresholded to remove background, pixel intensity was converted to a binary, and the images were segmented into objects; objects matching plaques on unmodified images were selected, and their area was quantified, as done previously (10).

### Statistical analysis

Statistical differences between means were determined with GraphPad Prism (version 10). Differences between the means of three or more groups were tested by either two-way ANOVA with Fisher’s or Holm-Sidak’s multiple comparisons test or one-way ANOVA with Dunnett’s multiple comparisons test. Differences between the means of two groups were tested by Student’s *t*-test unless otherwise noted.

## SUPPLEMENTAL MATERIAL

Supplemental Material mbio.01674-25-s0001.pdf Supplemental methods, references, figures, and tables.

ASM does not own the copyrights to Supplemental Material that may be linked to, or accessed through, an article. The authors have granted ASM a non-exclusive, world-wide license to publish the Supplemental Material files. Please contact the corresponding author directly for reuse.
