# Supplementary material for: A translocation-competent pore is required for Shigella flexneri to escape from the double membrane vacuole during intercellular spread
Source: mBio. 2025 Aug 11;16(9):e01674-25. doi: 10.1128/mbio.01674-25 (PMC12421969; doi:10.1128/mbio.01674-25)
Supplement: Supplemental Material — Supplemental methods, references, figures, and tables. [file mbio.01674-25-s0001.pdf]

A translocation-competent pore is required for *Shigella flexneri* to escape from the double membrane vacuole during intercellular spread.

Julie E. Raab,[a] Tucker B. Harju,[a] Jody D. Toperzer,[a] Jeffrey K. Duncan-Lowey [b, c], Connon I. Thomas [d], Anza Darehshouri [d], Marcia B. Goldberg [b, c], Brian C. Russo[a,#]

[a] Department of Immunology and Microbiology, University of Colorado - Anschutz Medical Campus, Aurora, Colorado, USA.

[b] Division of Infectious Diseases, Massachusetts General Hospital, Boston, Massachusetts, USA.

[c] Department of Microbiology, Blavatnik Institute, Harvard Medical School, Boston, Massachusetts, USA.

[d] Department of Cell and Developmental Biology, University of Colorado - Anschutz Medical Campus, Aurora, Colorado, USA.

Running Head: Translocation-competent pores enable *S.flexneri* spread

[#]Address correspondence to: Brian C. Russo, brian.russo@cuanschutz.edu.

SUPPLEMENTARY MATERIALS AND METHODS

Secretion Assays

T3SS secretion was measured as previously described[1-4]. Bacteria were cultured at 37°C with shaking at 250 rpm overnight, then back diluted, induced when indicated with 1.2% arabinose, and normalized by measuring optical density at 600 nm (OD_{600}). Bacteria were resuspended in phosphate-buffered saline (PBS) containing 1.2% arabinose, where appropriate, and 10 µM Congo red, and incubated for 2 hours in a 37°C water bath. Bacteria were pelleted at 15,000 x *g* and the supernatant collected. Western blots were used to detect IpaC and IpaC-FLAG37 in the pellets and supernatants.

Pore Formation by Erythrocyte Lysis Assay

Pore formation in sheep erythrocyte membranes was monitored by assessing the efficiency of erythrocyte lysis as done previously[2-5]. Briefly, defibrinated sheep erythrocytes (HemoStat) were pelleted at 2,000 x *g* and resuspended in 100 µL of PBS. Erythrocytes were infected at an MOI of 25 in 100 µl of PBS supplemented with 1.2% arabinose. Bacteria were centrifuged onto the erythrocytes at 2,000 x *g* for 10 min at 25°C and were co-cultured with the erythrocytes for 50 minutes at 37°C. The bacterial and erythrocyte cocultures were mixed by pipetting and then centrifuged again at 2,000 x *g* for 10 min at 25°C. As a positive control for lysis, an aliquot of uninfected erythrocytes was treated with 0.02% SDS. The supernatants were collected, and abundance of hemoglobin released was measured by absorbance at 570 nm using an Epoch II plate reader (BioTech) or Wallac 1420 Victor2 microplate reader (Perkin Elmer).

Translocation and Docking

The measurement of docking and T3SS activation was performed as previously described[2-4, 6]. Briefly, MEFs were seeded at 3×10^5 cells per well on coverslips in a six-well plate. Bacteria that constitutively produce mCherry under the *rpsM* promoter and harbor the TSAR reporter plasmid were grown to exponential phase and added to cells at an MOI of 200. The TSAR reporter, which expresses green fluorescent protein (GFP) when the bacterial effector OspD is secreted through

the T3SS[7], is an indicator of active T3SS secretion. Bacteria were then centrifuged onto cells at 800 x *g* for 10 minutes at 25°C. The co-culture was incubated at 37°C for an additional 50 minutes. The infected cells were washed with HBSS and fixed with 3.7% paraformaldehyde. DNA was stained with Hoechst. Coverslips were mounted onto glass slides with ProLong Diamond (Invitrogen). Bacteria were examined by fluorescence microscopy using a Nikon Eclipse TE-300 with appropriate filters. Bacterial docking was quantified by determining the number of mCherry-producing bacteria that remained associated with cells. Bacteria with active T3SS were determined by counting the number of cell-associated bacteria expressing GFP. For each condition of each independent experiment, 20 to 250 eukaryotic cells and 20 to 210 bacteria were analyzed.

SUPPLEMENTARY REFERENCES

1. Bahrani, F.K., Sansonetti, P.J. & Parsot, C. Secretion of Ipa proteins by Shigella flexneri: inducer molecules and kinetics of activation. *Infect Immun* **65**, 4005-4010 (1997).
2. Russo, B.C. *et al.* Intermediate filaments enable pathogen docking to trigger type 3 effector translocation. *Nat Microbiol* **1**, 16025 (2016).
3. Russo, B.C., Duncan, J.K. & Goldberg, M.B. Topological Analysis of the Type 3 Secretion System Translocon Pore Protein IpaC following Its Native Delivery to the Plasma Membrane during Infection. *mBio* **10** (2019).
4. Russo, B.C., Duncan-Lowey, J.K., Chen, P. & Goldberg, M.B. The type 3 secretion system requires actin polymerization to open translocon pores. *PLoS Pathog* **17**, e1009932 (2021).
5. Blocker, A. *et al.* The tripartite type III secreton of Shigella flexneri inserts IpaB and IpaC into host membranes. *J Cell Biol* **147**, 683-693 (1999).
6. Russo, B.C., Duncan, J.K., Wiscovitch, A.L., Hachey, A.C. & Goldberg, M.B. Activation of Shigella flexneri type 3 secretion requires a host-induced conformational change to the translocon pore. *PLoS Pathog* **15**, e1007928 (2019).
7. Campbell-Valois, F.X. *et al.* A fluorescent reporter reveals on/off regulation of the Shigella type III secretion apparatus during entry and cell-to-cell spread. *Cell Host Microbe* **15**, 177-189 (2014).

SUPPLEMENTARY FIGURE LEGENDS

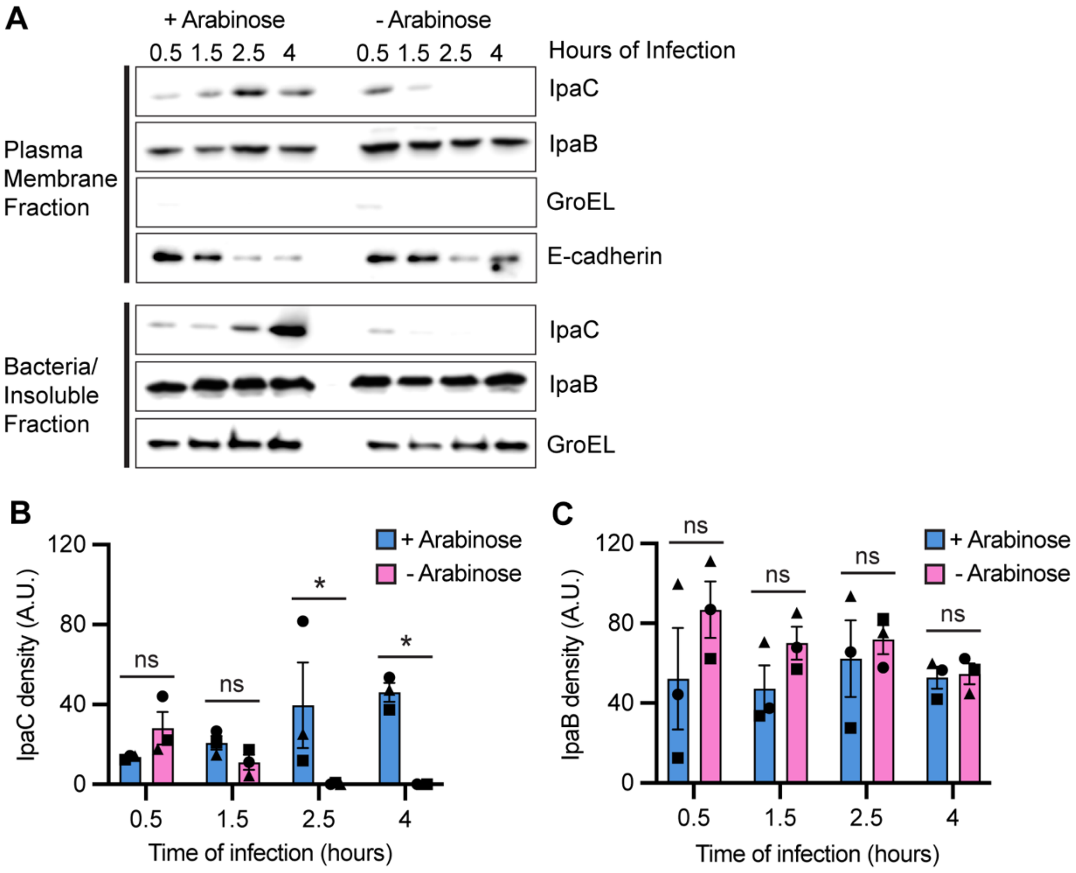

Figure S1: IpaC and IpaB are present in the host membrane at the time of bacterial spread in Caco-2 cells.

A) Representative western blots showing abundance of IpaC, IpaB, GroEL (bacterial cytoplasmic protein), and E-cadherin (host membrane protein) in the membrane fraction (top panels) and of IpaC, IpaB, and GroEL in the bacteria/insoluble fraction (bottom panels) from infected Caco-2 cells. **B-C)** Quantification of the amount of IpaC **(B)** or IpaB **(C)** in the membrane fraction with (light blue) or without (magenta) arabinose included in the media during infection to induce IpaC production. Three independent experiments were performed for each infection condition at each timepoint. Data are mean ± SEM, each experiment is matched by symbol. Mixed effects analysis with Fisher's multiple comparisons test. **$p<0.01$.

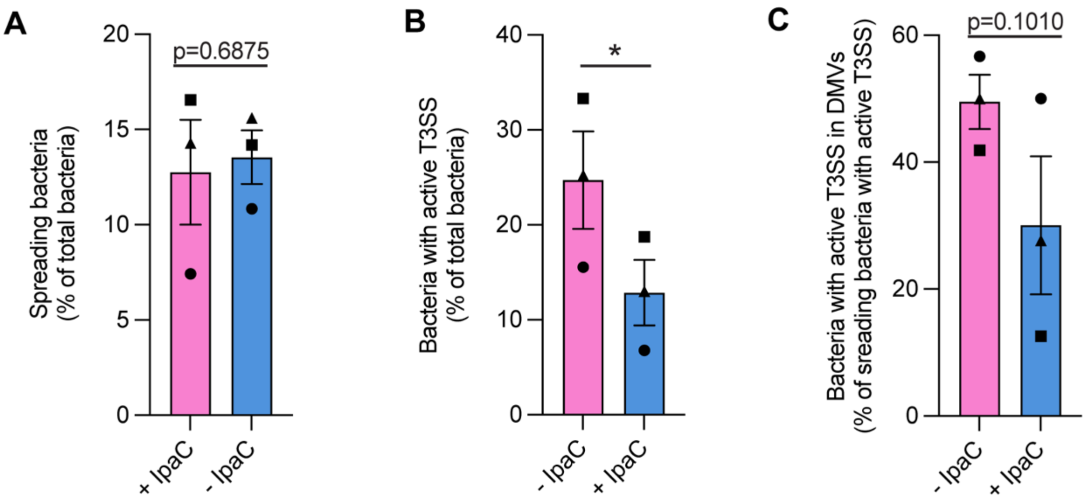

Figure S2: **Bacteria not producing IpaC during spread show more T3SS activation.**

From images represented in Figure 2: **A)** percentage of bacteria spreading at 4 hours of infection, **B)** percentage of bacteria with active T3SS, and **C)** percentage of bacteria that are spreading and in DMVs with active T3SS at 4 hours of infection. **A-C)** Bacteria producing (+ IpaC, aqua) or not producing (- IpaC, magenta) IpaC during infection. Data are mean ± SEM of three independent experiments, each experiment matched by symbol. *p<0.05, by paired t-test.

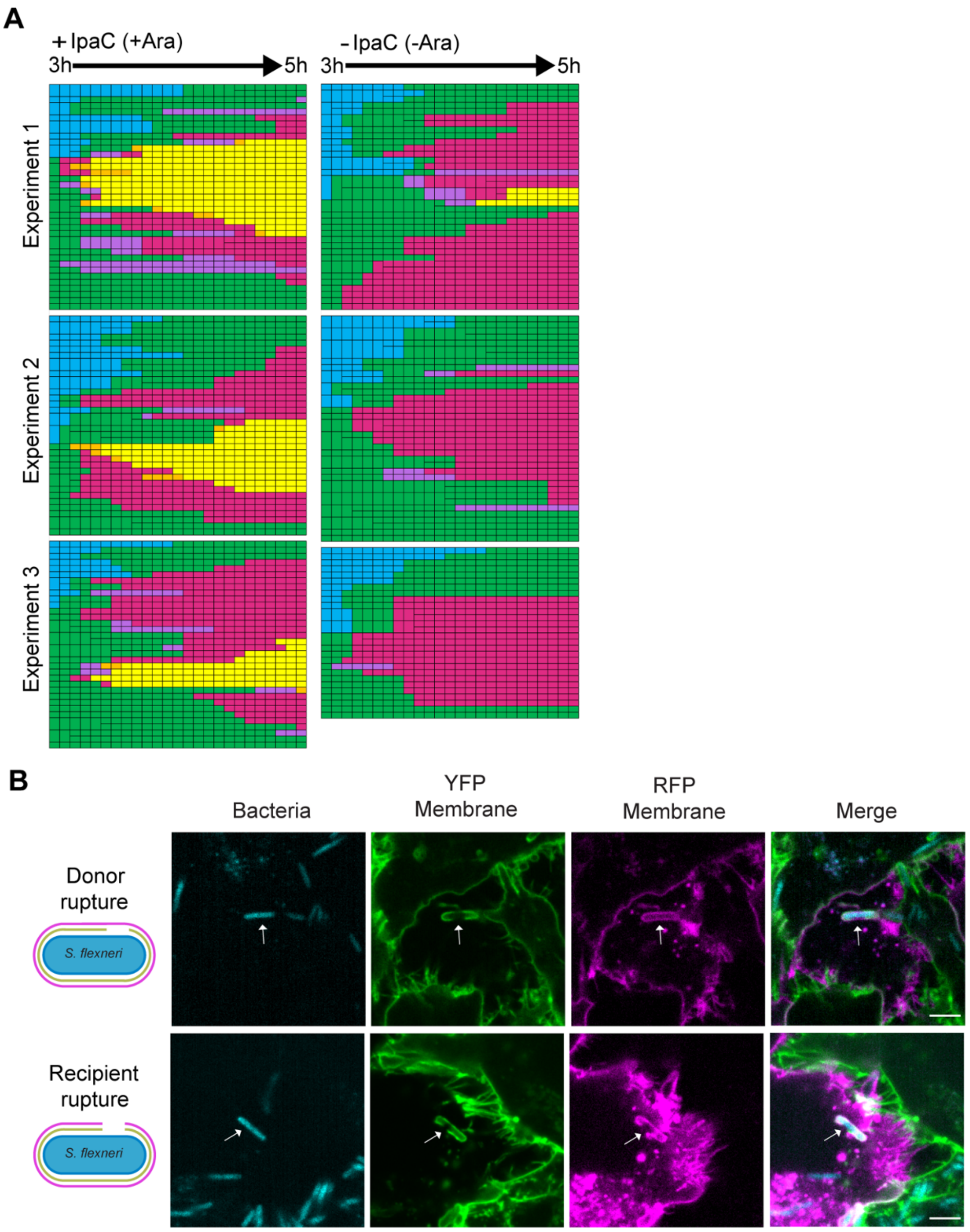

Figure S3: Tracking individual bacteria over time reveals DMV intermediates.

A) Three independent experiments tracking bacteria during spread as in Fig 4. Experiment 1 is reproduced in Fig 4C. **B)** Representative immunofluorescence images showing bacteria in a DMV intermediate with donor membrane rupture (top) or recipient membrane rupture (bottom). White

arrow, membrane rupture; cyan, bacteria; green, HeLa pmbYFP cell membranes; magenta, HeLa pmbRFP cell membranes; scale bar, 5 µm.

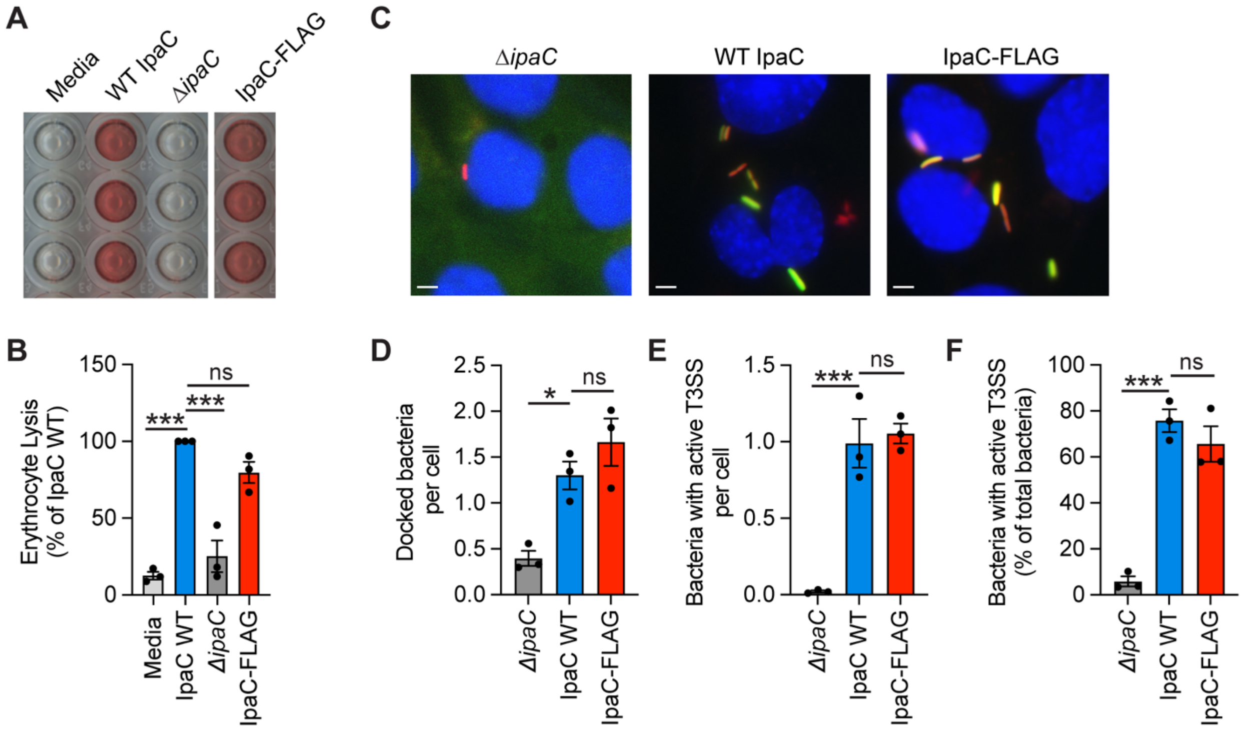

Figure S4: ***S. flexneri*** **producing IpaC-FLAG can form pores and translocate effectors.**

A) Representative image of hemoglobin released from erythrocytes. **B)** Quantification of hemoglobin released in experiments represented in A. Data points are individual experiments; data are the mean ± SEM of three experiments per strain. ns (not significant), ***p<0.001. One-way ANOVA with Dunnett's *post hoc* test. **C)** Representative images of infection of MEFs by bacteria producing no IpaC, WT IpaC, or IpaC-FLAG after 1 hour of infection. Red, bacteria; green, bacteria with active T3SS; blue, DNA; scale bar, 5 µm. **D-F)** Number of docked bacteria per cell **(D)**, number of bacteria with active T3SS secretion per cell **(E)**, and percentage of docked bacteria with activate T3SS secretion **(F)** in experiments represented in panel **C.** Data points are

individual experiments; data are the mean ± SEM of three experiments per strain. ns (not significant), *p<0.05, ***p<0.001. One-way ANOVA with Dunnett's multiple comparisons test.

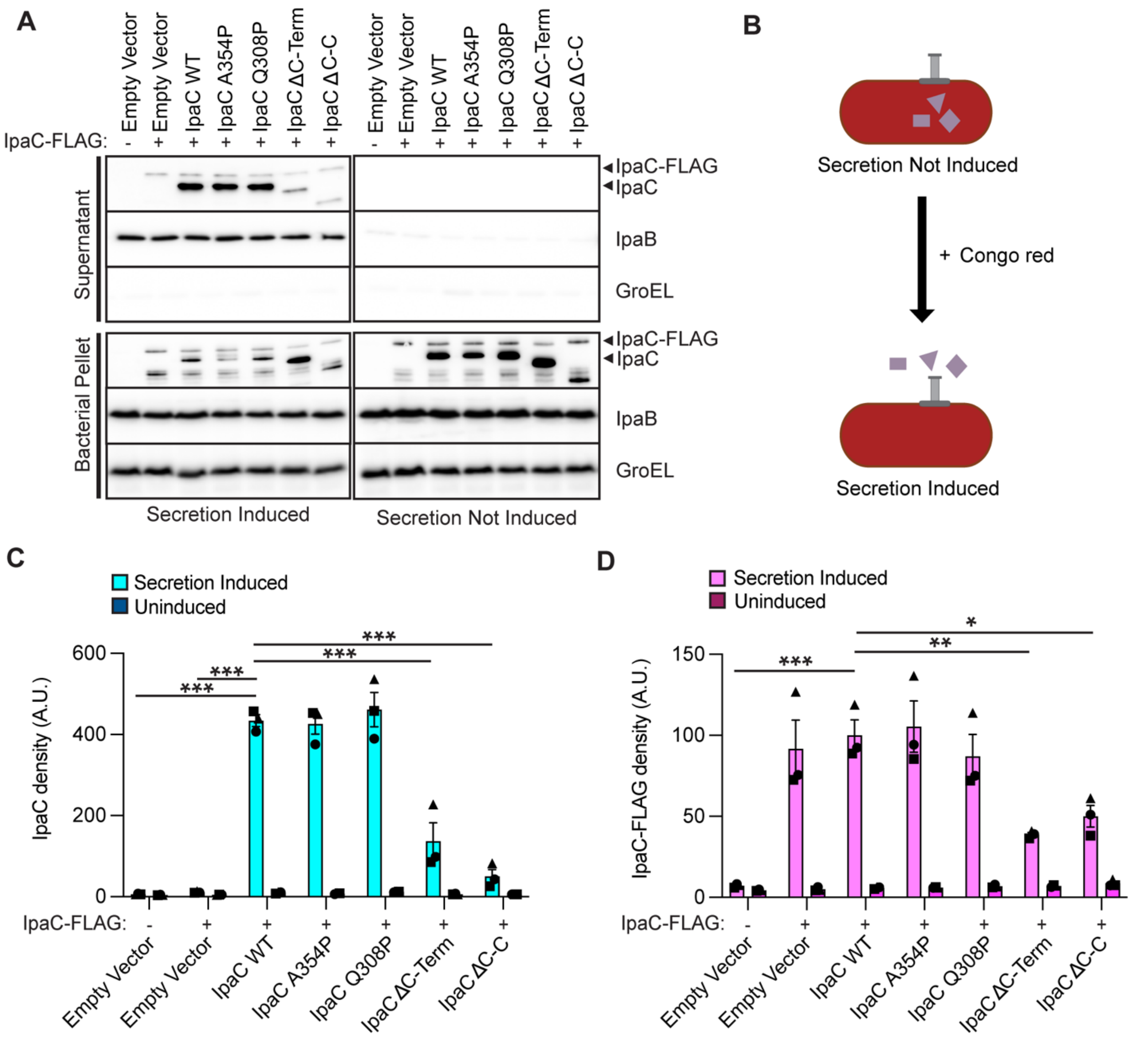

Figure S5: ***S. flexneri* producing IpaC variants and IpaC-FLAG can secrete both when induced.**

A) Representative western blots showing abundance of IpaC-FLAG (induced), IpaC variants, IpaB, and GroEL in the supernatant (top panels) and the bacterial pellet (bottom panels) following induction or not of secretion by Congo red. C-Term (C-terminus), C-C (Coiled-coil). **B)** Schematic

showing induction of secretion by Congo red. **C-D)** Quantification of the amount of IpaC variants **(C)** or IpaC-FLAG **(D)** in the supernatant when secretion is induced or uninduced. Data are mean ± SEM of three independent experiments, each experiment is matched by symbol. Two-way ANOVA with Dunnett's multiple comparisons test. ns (not significant), *p<0.05, **p<0.01, ***p<0.001.

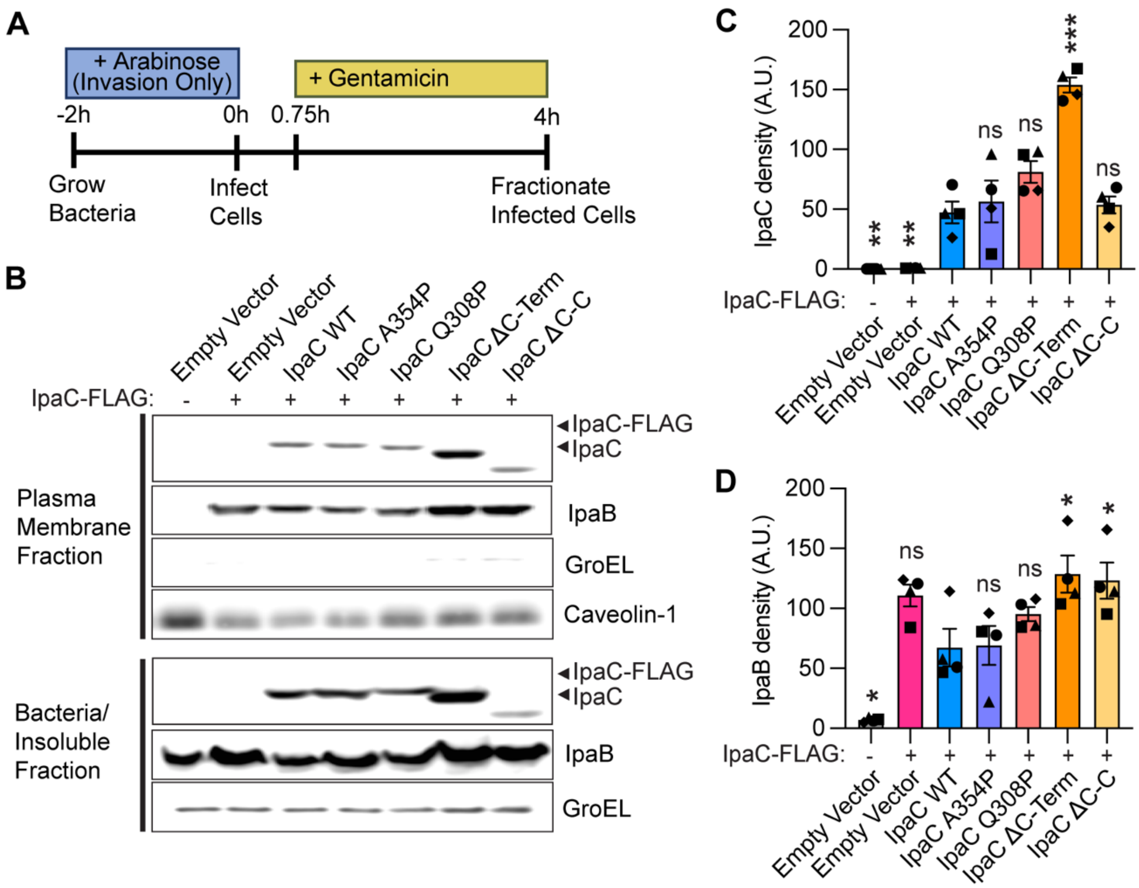

Figure S6: Insertion of pore proteins into the host membrane is not sufficient for spread. **A)** Schematic showing the experimental protocol. "Invasion Only" indicates arabinose was only present in the back dilution to induce the production of IpaC-FLAG to enable invasion. Gentamicin was added at 45 minutes of infection to kill extracellular bacteria. **B)** Representative western blots showing abundance of IpaC variants, IpaB, GroEL, and Caveolin-1 in the membrane fraction (top panels) and of IpaC, IpaB, and GroEL in the bacteria/insoluble fraction (bottom panels). IpaC-

FLAG was only induced before infection to enable invasion. C-Term (C-terminus), C-C (Coiled-coil). **C-D)** Quantitative measurements of IpaC **(C)** or IpaB **(D)** in the membrane fraction**.** Data are mean ± SEM of four independent experiments, each experiment matched by symbol. ns (not significant), *p<0.05, **p<0.01, ***p<0.001 by one-way ANOVA with Dunnett's multiple comparisons test.

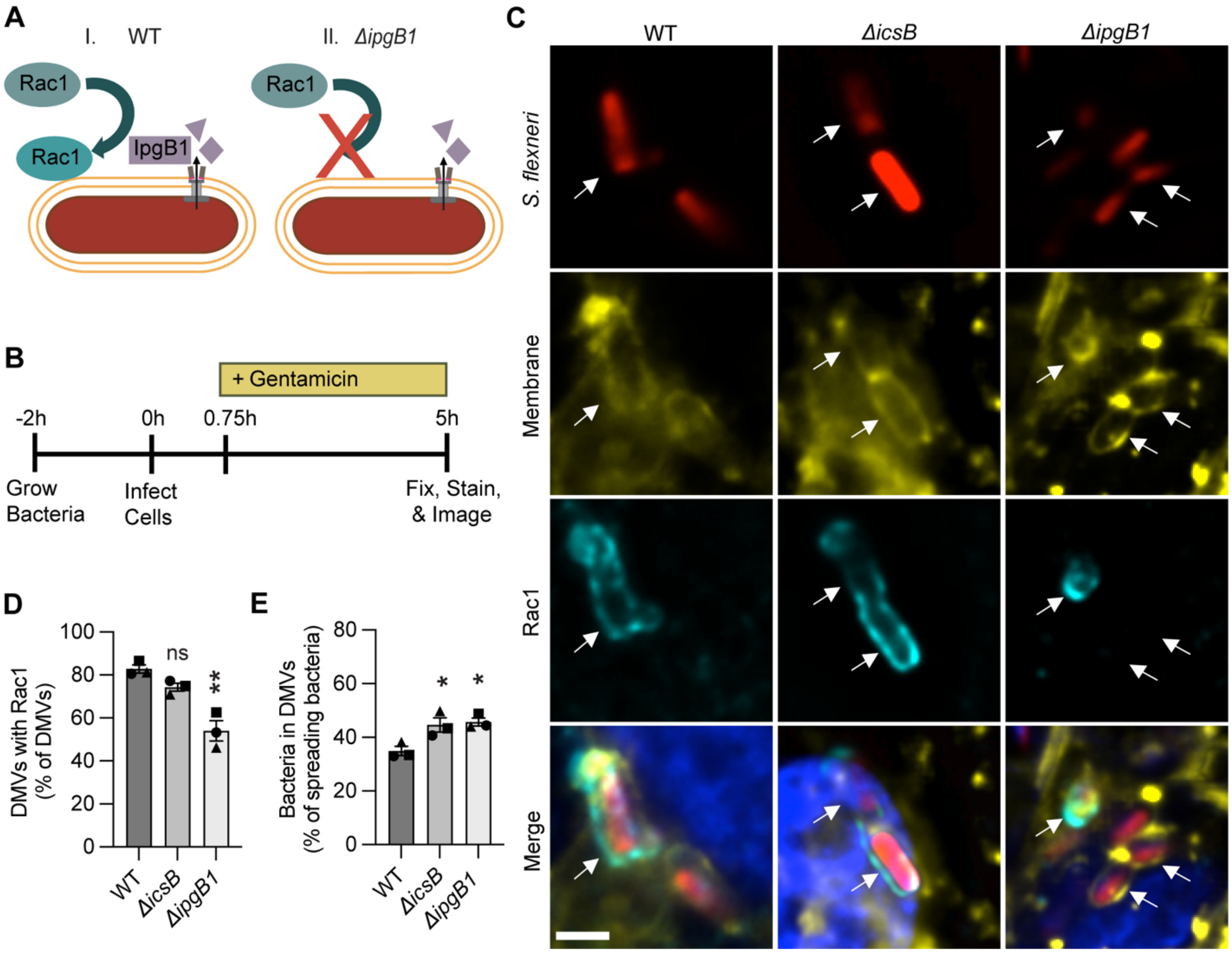

Figure S7: Rac1 colocalization with DMVs is associated with IpgB1 presence.

A) Schematic displaying **I.** IpgB1 recruits Rac1 to a DMV. **II.** In the absence of IpgB1, Rac1 is not recruited to a DMV. **B)** Schematic showing the experimental protocol. Gentamicin was added at 45 minutes of infection to kill extracellular bacteria. **C)** Representative immunofluorescence images of HT-29 pmbYFP cells infected with bacteria producing indicated *S. flexneri* knockout at

5 hours of infection. Bacteria in a DMV (white arrow). Red, bacteria; yellow, HT-29 cell membranes; cyan, Rac1; blue, DNA; scale bar, 2 μm. **D-E)** From images represented in **C,** percent of bacteria within DMVs that colocalize with Rac1 **(D)**, and percent of bacteria that are spreading and in DMVs **(E)**. Data are mean ± SEM of three independent experiments, each experiment is matched by symbol. ns (not significant), *p<0.05, **p<0.01 by one-way ANOVA with Dunnett's multiple comparisons test.

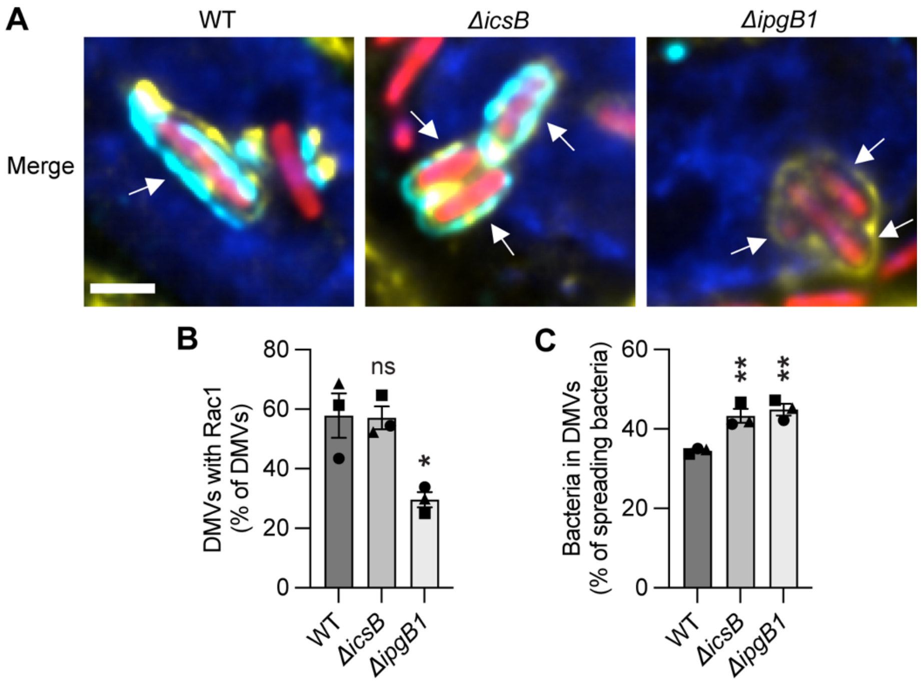

Figure S8: Rac1 colocalization with DMVs is associated with IpgB1 presence in HeLa cells. **A)** Representative immunofluorescence images of HeLa pmbYFP cells infected with bacteria producing indicated *S. flexneri* knockout at 4 hours of infection. Bacteria within a DMV (white arrow). Red, bacteria; yellow, HeLa cell membranes; cyan, Rac1; blue, DNA; scale bar, 2 μm. **B-C)** From images represented in **A,** percent of bacteria within DMVs that colocalize with Rac1 **(B)**, and percent of bacteria that are spreading and in DMVs **(C)**. Data are mean ± SEM of three independent experiments, each experiment is matched by symbol. ns (not significant), *p<0.05, **p<0.01 by one-way ANOVA with Dunnett's multiple comparisons test.

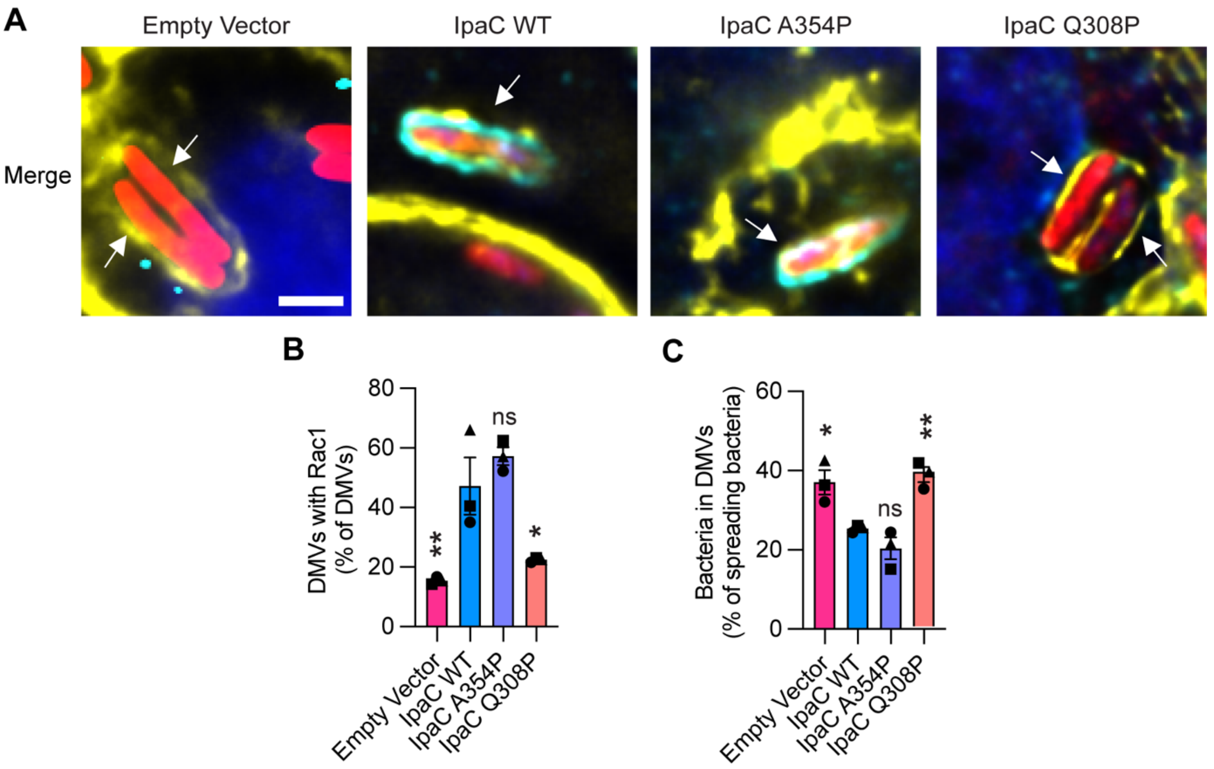

Figure S9: **Translocation-competent pores are required for *S. flexneri* to recruit Rac1 and to escape from DMVs in HeLa cells.**

A) Representative immunofluorescence images of HeLa pmbYFP cells infected with bacteria producing indicated IpaC variants at 4 hours of infection. All strains included IpaC-FLAG and were induced only before infection to enable invasion. Bacteria within a DMV (white arrow). Red, bacteria; yellow, HeLa cell membranes; cyan, Rac1; blue, DNA; scale bar, 2 µm. **B-C)** From images represented in **A,** percent of bacteria within DMVs that colocalize with Rac1 **(B)**, and percent of bacteria that are spreading and in DMVs **(C)**. Data are mean ± SEM of three independent experiments, each experiment is matched by symbol. ns (not significant), *p<0.05, **p<0.01 by one-way ANOVA with Dunnett's multiple comparisons test.

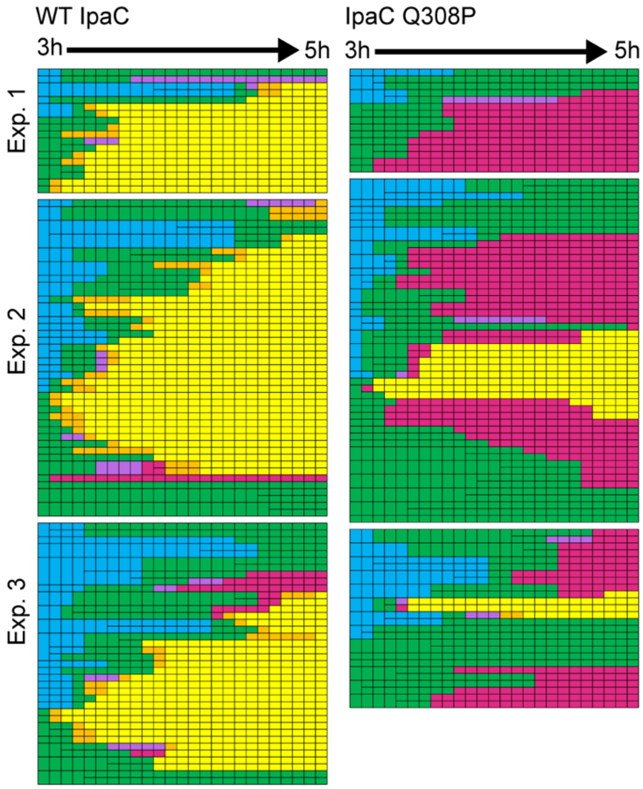

Figure S10: All tracking of individual bacteria from Figure 7.

Three independent experiments tracking bacteria during spread as in Figure 7. Exp. 2 is reproduced in Fig 7F.

	Total bacteria		Spreading bacteria (% of total bacteria)		Bacteria in DMVs (% of total bacteria)		Total bacteria in DMVs (% of spreading bacteria)		Bacteria with active T3SS (% of total bacteria)		Spreading bacteria with active T3SS (% of bacteria with active T3SS)		Bacteria with active T3SS in DMVs (% of bacteria with active T3SS)		Bacteria with active T3SS in DMVs (% of spreading bacteria with active T3SS)	
	**- IpaC**	**+ IpaC**	**- IpaC**	**+ IpaC**	**- IpaC**	**+ IpaC**	**- IpaC**	**+ IpaC**	**- IpaC**	**+ IpaC**	**- IpaC**	**+ IpaC**	**- IpaC**	**+ IpaC**	**- IpaC**	**+ IpaC**
Experiment 1	765	942	83 (10.9%)	70 (7.4%)	32 (4.2%)	22 (2.3%)	32 (38.6%)	22 (31.4%)	119 (15.6%)	64 (6.8%)	30 (36.1%)	28 (40.0%)	17 (14.3%)	14 (21.9%)	17 (56.7%)	14 (50.0%)
Experiment 2	2755	2330	391 (14.2%)	386 (16.6%)	129 (4.7%)	75 (3.2%)	129 (33.0%)	75 (19.4%)	918 (33.3%)	437 (18.8%)	172 (44.0%)	223 (57.8%)	72 (7.8%)	28 (6.4%)	72 (41.9%)	28 (12.6%)
Experiment 3	1192	1260	186 (15.6%)	180 (14.3%)	77 (6.5%)	50 (4.0%)	77 (41.4%)	50 (24.8%)	301 (25.3%)	164 (13.0%)	52 (28.0%)	47 (26.1%)	26 (8.6%)	13 (7.9%)	26 (50.0%)	13 (27.7%)

Table S1: Quantifications of bacteria from Figure 2.

Quantification of images acquired across three independent experiments at 4 hours of infection in Figure 2 for bacteria not producing (- IpaC, pink) or producing (+ IpaC, blue) IpaC during infection. Total bacteria: all mCherry+ bacteria. Spreading bacteria: all mCherry+ bacteria observed in protrusions or DMVs. Bacteria in DMVs: all mCherry+ bacteria observed in DMVs. Bacteria with active T3SS: mCherry+ bacteria that are also GFP+. Spreading bacteria with active T3SS: Bacteria with active T3SS observed in protrusions or DMVs. Bacteria with active T3SS in DMVs: Bacteria with active T3SS observed in DMVs.

	Bacteria spreading between YFP and RFP cells		Bacteria in DMVs (% of spreading bacteria)		Bacteria in DMVs with 2 intact membranes (% of bacteria in DMVs)		Bacteria in DMVs with 1 intact membrane (% of bacteria in DMVs)	
	**- IpaC**	**+ IpaC**	**- IpaC**	**+ IpaC**	**- IpaC**	**+ IpaC**	**- IpaC**	**+ IpaC**
Experiment 1	144	307	56 (38.9%)	92 (30.0%)	43 (76.8%)	78 (84.8%)	13 (23.2%)	14 (15.2%)
Experiment 2	293	654	114 (38.9%)	206 (31.5%)	94 (82.5%)	165 (80.1%)	20 (17.5%)	41 (19.9%)
Experiment 3	297	540	119 (40.1%)	136 (25.2%)	95 (79.8%)	101 (74.3%)	24 (20.2%)	35 (25.7%)

Table S2: Quantifications of bacteria from Figure 3.

Quantification of images acquired across three independent experiments at 4 hours of infection in Figure 3 for bacteria not producing (- IpaC, pink) or producing (+ IpaC, blue) IpaC during infection. Bacteria spreading between YFP and RFP cells: all bacteria observed in protrusions or DMVs that originated from a cell with the other membrane-anchored fluorescent protein. Bacteria in DMVs: all bacteria observed in DMVs with either 1 or 2 intact membranes. Bacteria in DMVs with two intact membranes: bacteria in DMVs that have an RFP membrane and a YFP membrane. Bacteria in DMVs with one intact membrane: bacteria in DMVs that have visible either an RFP membrane or a YFP membrane and originated from a cell with the other membrane-anchored fluorescent protein.

	Total bacteria			Spreading bacteria (% of total bacteria)			Bacteria in DMVs (% of total bacteria)			Bacteria in DMVs (% of spreading bacteria)			DMVs with Rac1 (% of DMVs)		
	WT	*ΔicsB*	*ΔipgB1*	WT	*ΔicsB*	*ΔipgB1*	WT	*ΔicsB*	*ΔipgB1*	WT	*ΔicsB*	*ΔipgB1*	WT	*ΔicsB*	*ΔipgB1*
Experiment 1	495	1172	527	109 (22.0%)	248 (21.2%)	169 (32.1%)	36 (7.3%)	101 (8.6%)	75 (14.2%)	36 (33.0%)	101 (40.7%)	75 (44.4%)	29 (80.6%)	78 (77.2%)	40 (53.3%)
Experiment 2	1018	1364	1887	245 (24.1%)	311 (22.8%)	367 (19.4%)	82 (8.1%)	135 (9.9%)	179 (9.5%)	82 (33.5%)	135 (43.4%)	179 (48.8%)	71 (86.6%)	101 (74.8%)	112 (62.6%)
Experiment 3	1680	1523	829	392 (23.3%)	187 (12.3%)	182 (22.0%)	150 (8.9%)	93 (6.1%)	80 (9.7%)	150 (38.2%)	93 (49.7%)	80 (44.0%)	122 (81.3%)	66 (71.0%)	37 (46.3%)

Table S3: Quantifications of bacteria from infected HT-29 cells in Figure S7.

Quantification of images acquired across three independent experiments at 5 hours of infection in Figure S7 for indicated *S. flexneri* strains. Total bacteria: all mCherry+ bacteria. Spreading bacteria: all mCherry+ bacteria observed in protrusions or DMVs. Bacteria in DMVs: all mCherry+ bacteria observed in DMVs. Bacteria in DMVs with Rac1: all mCherry+ bacteria observed in DMVs that also colocalize with Rac1.

	Total bacteria			Spreading bacteria (% of total bacteria)			Bacteria in DMVs (% of total bacteria)			Bacteria in DMVs (% of spreading bacteria)			DMVs with Rac1 (% of DMVs)		
	WT	*ΔicsB*	*ΔipgB1*	WT	*ΔicsB*	*ΔipgB1*	WT	*ΔicsB*	*ΔipgB1*	WT	*ΔicsB*	*ΔipgB1*	WT	*ΔicsB*	*ΔipgB1*
Experiment 1	352	860	492	131 (37.2%)	165 (19.2%)	154 (31.3%)	46 (13.1%)	68 (7.9%)	65 (13.2%)	46 (35.1%)	68 (41.2%)	65 (42.2%)	20 (43.5%)	37 (54.4%)	22 (33.8%)
Experiment 2	1013	855	713	169 (16.7%)	109 (12.7%)	144 (20.2%)	57 (5.6%)	51 (6.0%)	68 (9.5%)	57 (33.7%)	51 (46.8%)	68 (47.2%)	35 (61.4%)	33 (64.7%)	17 (25.0%)
Experiment 3	1904	1540	1623	247 (13.0%)	210 (13.6%)	325 (20.0%)	86 (4.5%)	88 (5.7%)	147 (9.1%)	86 (34.8%)	88 (41.9%)	147 (45.2%)	59 (68.6%)	46 (52.3%)	44 (29.9%)

Table S4: Quantifications of bacteria from infected HeLa cells in Figure S8.

Quantification of images acquired across three independent experiments at 4 hours of infection in Figure S8 for indicated *S. flexneri* strains. Total bacteria: all mCherry+ bacteria. Spreading bacteria: all mCherry+ bacteria observed in protrusions or DMVs. Bacteria in DMVs: all mCherry+ bacteria observed in DMVs. Bacteria in DMVs with Rac1: all mCherry+ bacteria observed in DMVs that also colocalize with Rac1.

	Total bacteria				Spreading bacteria (% of total bacteria)				Bacteria in DMVs (% of total bacteria)				Bacteria in DMVs (% of spreading bacteria)				DMVs with Rac1 (% of DMVs)			
	Empty Vector	IpaC WT	IpaC A354P	IpaC Q308P	Empty Vector	IpaC WT	IpaC A354P	IpaC Q308P	Empty Vector	IpaC WT	IpaC A354P	IpaC Q308P	Empty Vector	IpaC WT	IpaC A354P	IpaC Q308P	Empty Vector	IpaC WT	IpaC A354P	IpaC Q308P
Experiment 1	202	522	716	124	94 (46.5%)	90 (17.2%)	183 (25.6%)	49 (39.5%)	57 (28.2%)	31 (5.9%)	64 (8.9%)	25 (20.2%)	57 (60.6%)	31 (34.4%)	64 (35.0%)	25 (51.0%)	2 (3.5%)	15 (48.4%)	47 (73.4%)	0 (0.0%)
Experiment 2	237	943	340	176	87 (36.7%)	168 (17.8%)	93 (27.4%)	60 (34.1%)	40 (16.9%)	48 (5.1%)	25 (7.4%)	26 (14.8%)	40 (46.0%)	48 (28.6%)	25 (26.9%)	26 (43.3%)	7 (17.5%)	37 (77.1%)	21 (84.0%)	8 (30.8%)
Experiment 3	421	475	631	332	114 (27.1%)	101 (21.3%)	162 (25.7%)	110 (33.1%)	56 (13.3%)	29 (6.1%)	60 (9.5%)	59 (17.8%)	56 (49.1%)	29 (28.7%)	60 (37.0%)	59 (53.6%)	5 (8.9%)	23 (79.3%)	42 (70.0%)	7 (11.9%)

Table S5: Quantifications of bacteria from infected HT-29 cells in Figure 6.

Quantification of images acquired across three independent experiments at 5 hours of infection in Figure 6 for *S. flexneri* producing indicated IpaC variant. Total bacteria: all mCherry+ bacteria. Spreading bacteria: all mCherry+ bacteria observed in protrusions or DMVs. Bacteria in DMVs: all mCherry+ bacteria observed in DMVs. Bacteria in DMVs with Rac1: all mCherry+ bacteria observed in DMVs that also colocalize with Rac1.

	Total bacteria				Spreading bacteria (% of total bacteria)				Bacteria in DMVs (% of total bacteria)				Bacteria in DMVs (% of spreading bacteria)				DMVs with Rac1 (% of DMVs)			
	Empty Vector	IpaC WT	IpaC A354P	IpaC Q308P	Empty Vector	IpaC WT	IpaC A354P	IpaC Q308P	Empty Vector	IpaC WT	IpaC A354P	IpaC Q308P	Empty Vector	IpaC WT	IpaC A354P	IpaC Q308P	Empty Vector	IpaC WT	IpaC A354P	IpaC Q308P
Experiment 1	883	722	1715	966	56 (6.3%)	82 (11.4%)	94 (5.5%)	144 (14.9%)	18 (2.0%)	20 (2.8%)	23 (1.3%)	51 (5.3%)	18 (32.1%)	20 (24.4%)	23 (24.5%)	51 (35.4%)	3 (16.7%)	7 (35.0%)	12 (52.2%)	11 (21.6%)
Experiment 2	299	446	433	268	77 (25.8%)	161 (36.1%)	138 (31.9%)	124 (46.3%)	28 (9.4%)	42 (9.4%)	32 (7.4%)	52 (19.4%)	28 (36.4%)	42 (26.1%)	32 (23.2%)	52 (41.9%)	4 (14.3%)	17 (40.5%)	20 (62.5%)	12 (23.1%)
Experiment 3	286	1155	1297	185	61 (21.3%)	242 (21.0%)	130 (10.0%)	78 (42.1%)	26 (9.1%)	62 (5.4%)	28 (2.2%)	31 (16.8%)	26 (42.6%)	62 (25.6%)	28 (21.5%)	31 (39.7%)	4 (15.4%)	41 (66.1%)	16 (57.1%)	7 (22.6%)

Table S6: Quantifications of bacteria from infected HeLa cells in Figure S9.

Quantification of images acquired across three independent experiments at 4 hours of infection in Figure S9 for *S. flexneri* producing indicated IpaC variant. Total bacteria: all mCherry+ bacteria. Spreading bacteria: all mCherry+ bacteria observed in protrusions or DMVs. Bacteria in DMVs: all mCherry+ bacteria observed in DMVs. Bacteria in DMVs with Rac1: all mCherry+ bacteria observed in DMVs that also colocalize with Rac1.

	Bacteria spreading between YFP and RFP cells		Bacteria in DMVs (% of spreading bacteria)		Bacteria in DMVs with 2 intact membranes (% of DMVs)		Bacteria in DMVs with 1 intact membrane (% of DMV)	
	**WT**	**Q308P**	**WT**	**Q308P**	**WT**	**Q308P**	**WT**	**Q308P**
Experiment 1	133	84	38 (28.6%)	34 (40.5%)	31 (81.6%)	26 (76.5%)	7 (18.4%)	8 (23.5%)
Experiment 2	171	79	49 (28.7%)	33 (41.8%)	40 (81.6%)	25 (75.8%)	9 (18.4%)	8 (24.2%)
Experiment 3	133	125	36 (27.1%)	56 (44.8%)	30 (83.3%)	45 (80.4%)	6 (16.7%)	11 (19.6%)

Table S7: Quantifications of bacteria from Figure 7.

Quantification of images acquired across three independent experiments at 4 hours of infection in Figure 7A-C for bacteria producing IpaC WT (blue) or IpaC Q308P (pink) during infection. Bacteria spreading between YFP and RFP cells: all bacteria observed in protrusions or DMVs that originated from a cell with the other membrane-anchored fluorescent protein. Bacteria in DMVs: all bacteria observed in DMVs with either 1 or 2 intact membranes. Bacteria in DMVs with two intact membranes: bacteria in DMVs that have an RFP membrane and a YFP membrane. Bacteria in DMVs with one intact membrane: bacteria in DMVs that have visible either an RFP membrane or a YFP membrane and originated from a cell with the other membrane-anchored fluorescent protein.
